# Growth-rate coordination across the width of a leaf preserves its flatness

**DOI:** 10.1371/journal.pbio.3003993

**Published:** 2026-09-08

**Authors:** Kate Harline, Brendan Lane, Maura J. Zimmermann, Antoine Fruleux, Gabriella Mosca, Sören Strauss, Nik Tavakolian, James W. Satterlee, Chun-Biu Li, Abhyudai Singh, Arezki Boudaoud, Richard S. Smith, Adrienne H. K. Roeder

**Affiliations:** 1 Weill Institute for Cell and Molecular Biology, Cornell University, Ithaca, New York, United States of America; 2 Section of Plant Biology, School of Integrative Plant Sciences, Cornell University, Ithaca, New York, United States of America; 3 Department of Computational and Systems Biology, John Innes Centre, Norwich Research Park, Norwich, United Kingdom; 4 LPTMS, CNRS, Université Paris-Saclay, Orsay, France; 5 Center for Plant Molecular Biology, University of Tübingen, Tübingen, Germany; 6 Cluster of Excellence GreenRobust, University of Tübingen, Tübingen, Germany; 7 Department of Comparative Development and Genetics, Max Planck Institute for Plant Breeding Research, Cologne, Germany; 8 Department of Mathematics, Stockholm University, Stockholm, Sweden; 9 Department of Electrical and Computer Engineering, Biomedical Engineering, University of Delaware, Newark, Delaware, United States of America; 10 LadHyX, CNRS, Ecole Polytechnique, IP Paris, Palaiseau, France; University of Cambridge, UNITED KINGDOM OF GREAT BRITAIN AND NORTHERN IRELAND

## Abstract

The growth and division of cells in plant leaves is highly dynamic in time and space, even though cells cannot move relative to their neighbors. Thus, organ shape must emerge from carefully coordinated growth, especially in leaves that remain relatively flat as they grow. Here we explored the phenotype of the *jagged and wavy* (*jaw-D*) mutant in *Arabidopsis thaliana*, in which the leaves do not remain flat. It has previously been shown that the *jaw-D* mutant phenotype is caused by the overexpression of *miR319*, which represses TCP transcription factors, thus delaying maturation of the leaf. We analyzed cell dynamics in wild type and *jaw-D* by performing time-lapse live imaging of developing leaves. We found that the progression of maturation from the tip of the leaf downward was delayed in *jaw-D* relative to wild type based on several markers of maturation, in agreement with the role of TCP transcription factors in promoting maturation. We further found that these changes in maturation were accompanied by differences in the coordination of growth across the leaf, particularly across the mediolateral axis, causing growth conflicts that prevent the leaf from remaining flat. Modeling revealed that curvature develops when growth is uneven across the leaf in the direction perpendicular to the direction of growth. Although leaf flatness is often framed as a problem that requires the local synchronization of growth on the abaxial versus adaxial sides (bottom versus top) of the leaf, our results based on the *jaw-D* phenotype suggest that wild-type plants also need to coordinate growth more globally across the leaf blade to maintain flatness.

## Abstract 2

The growth of plant leaves is highly dynamic and should be coordinated, but the mechanisms that preserve leaf flatness remain unclear. This study shows that global coordination of growth across the leaf blade is required to maintain flatness in Arabidopsis.

## Introduction

In nature, leaves exhibit a myriad of shapes and sizes. Yet, all leaves initially begin as a few cells recruited from the shoot apical meristem (SAM) [1–5]. Understanding how these few initial cells create recognizable forms in nature requires a combined exploration of growth dynamics, tissue mechanics, and genetic regulatory pathways. Imaging at multiple scales has revealed that development and growth in leaves are highly spatially and temporally dynamic [6–11]. In many species, including *Arabidopsis thaliana*, leaf development is characterized by a dynamic transition from a rapid to slow relative growth rate known as the basipetal growth gradient [7,11–13]. As the leaf expands, the relative growth rate first slows at the distal tip, and this zone of reduced growth progressively advances toward the proximal base. The decline in cell growth coincides with a basipetally moving wave of cellular differentiation and exit from proliferation [14]. Over developmental time, two coordinated processes occur simultaneously: (1) the wave of slowing growth and differentiation progresses from distal tip to proximal base, and (2) overall growth rates gradually decrease throughout the leaf, including within the faster-growing basal region [12]. At any given time point, these dynamics produce a spatial gradient in which cells at the distal tip grow more slowly than cells nearer the base. We therefore use the term basipetal growth gradient to refer to this integrated spatiotemporal pattern of growth deceleration and differentiation during leaf development.

Biomechanical principles predict that juxtaposition of different growth rates within a tissue can create growth conflicts, resulting in deformation of the tissue, including leaf curling and ruffling [15–18]. There are two major types of growth conflict: (1) growth conflict within a tissue layer, and (2) growth conflicts between two cell layers of a tissue, such as adaxial (top) and abaxial (bottom) sides. These two growth conflicts occur when cells within a tissue grow at a higher rate or in a different direction than neighboring cells, and the region is compressed by the neighborhood [19]. The compressive stress causes the tissue to buckle and either bend upward or downward. Loss of coordination of growth between the adaxial and abaxial sides of the sepal can cause the tissue to curve or, in more extreme cases, to make multiple complex ridges through buckling [20,21]. Buckling induced by internal growth conflicts has been used to explain the three-dimensional form of fronds in kelp [22], petals in the snapdragon [23], or wing imaginal discs in the fruit fly [24]. At a smaller scale, mechanical stress causes the cuticle at the base of the hibiscus petal to buckle, forming micro-ridges that generate iridescence [25–28]. Yet, leaves are usually flat, which is optimal for their physiological functions of photosynthesis and gas exchange [1]. It has been shown that a thin elastic sheet growing in three-dimensional space undergoes buckling and loses flatness if heterogeneity in growth is larger than a threshold, which may lead to fractal-like three-dimensional shapes [17,29–32], as observed for leaves of ornamental cabbage. Thus, it is not well understood how leaves maintain their flat form during development.

The *jagged and wavy* (*jaw-D*) mutant is one of the classic *Arabidopsis thaliana* mutants that disrupt leaf flatness [33,34]. *jaw-D* exhibits curly and wavy leaves with ruffles along the edges. The *jaw-D* wavy leaf phenotype has previously been attributed to extended cell division from an overactive marginal meristem [7,35–38]. *jaw-D* was discovered in an activation tagging screen, and the phenotype is caused by overexpression of the *microRNA319* (*miR319*) [33,34]. Over the last 20 years, the molecular mechanism through which miR319 regulates its targets has been worked out in exquisite detail; however, it is still not well understood how this pathway contributes to leaf flatness. miR319 targets the class II *teosinte branched 1, cycloidea, proliferating cell nuclear antigen factor 1 and 2 (TCP)* transcription factors which have been shown to repress growth and activate tissue differentiation [36,37,39,40]. miR319 targets, especially *TCP4,* are thought to regulate cell growth and differentiation through *cup-shaped cotyledons 2 (CUC2)* and the *CUC* regulator miR164 as well as auxin and cytokinin networks [41–45]. Based on GUS assays, previous studies have shown that the promoters of genes in the miR319 family drive expression at the base of leaves (miR319c) and floral organs (miR319a,c) [44,46]. Thus, the evidence suggests that miR319 and TCPs contribute to the basipetal growth gradient. Still, it is unclear how this dynamic basipetal growth gradient relates to maintenance of leaf flatness.

The regulation of TCPs by miR319 is well-conserved within angiosperms [47,48]. Therefore, this pathway could have broad implications for the flattening of leaves throughout the angiosperms. Notable homologs in other model species include the class II- defining member *cincinnata (CIN)* in snapdragon and *Lanceolate* in tomato [49–51], both of which when mutated or downregulated yield rippling and undulating leaves like the Arabidopsis *jaw-D* mutant*.* Increased curvature in *cin* leaves was attributed to decreased perception of a tip-derived differentiation signal and thus an escape from leaf margin growth regulation [49]. Still, the connection between organ shape and a proximal–distal differentiation wave regulating cell growth remains to be resolved.

To understand how leaf flatness emerges, we have tracked how cell growth, cell division and cell maturation give rise to the bottom (abaxial) epidermis of the developing leaf blade in living wild-type and *jaw-D* mutant first leaves. Because *jaw-D* overexpresses miR319, we expected to see a disruption of the basipetal growth gradient. We confirmed this disruption, finding that, at every time point, growth and cell division in *jaw-D* were shifted farther distally compared with wild type. In addition, the overall cell growth rates in *jaw-D* reached a lower maximum value than those in wild type. Although we did not observe overgrowth of the leaf margins in *jaw-D* relative to wild type in the first leaves, which was expected based on previous work, we did observe that cell growth across the mediolateral axis of the leaf was less coordinated and patchier than in wild type. In particular, growth was coordinated between the midrib and leaf blade in wild type, but less so in *jaw-D*. Modeling revealed that coordination of growth along the mediolateral axis, while still maintaining the growth gradient from tip to base, was sufficient to create a flat leaf blade, similar to wild type. In the model, lack of coordination of growth between midrib and blade created a curved leaf similar to *jaw-D*. Thus, our results suggest wild-type plants coordinate growth across the mediolateral axis of the leaf blade to maintain flatness.

## Results

### The generation of leaf shape can be captured through live imaging early stages of leaf development

**Fig 1:**
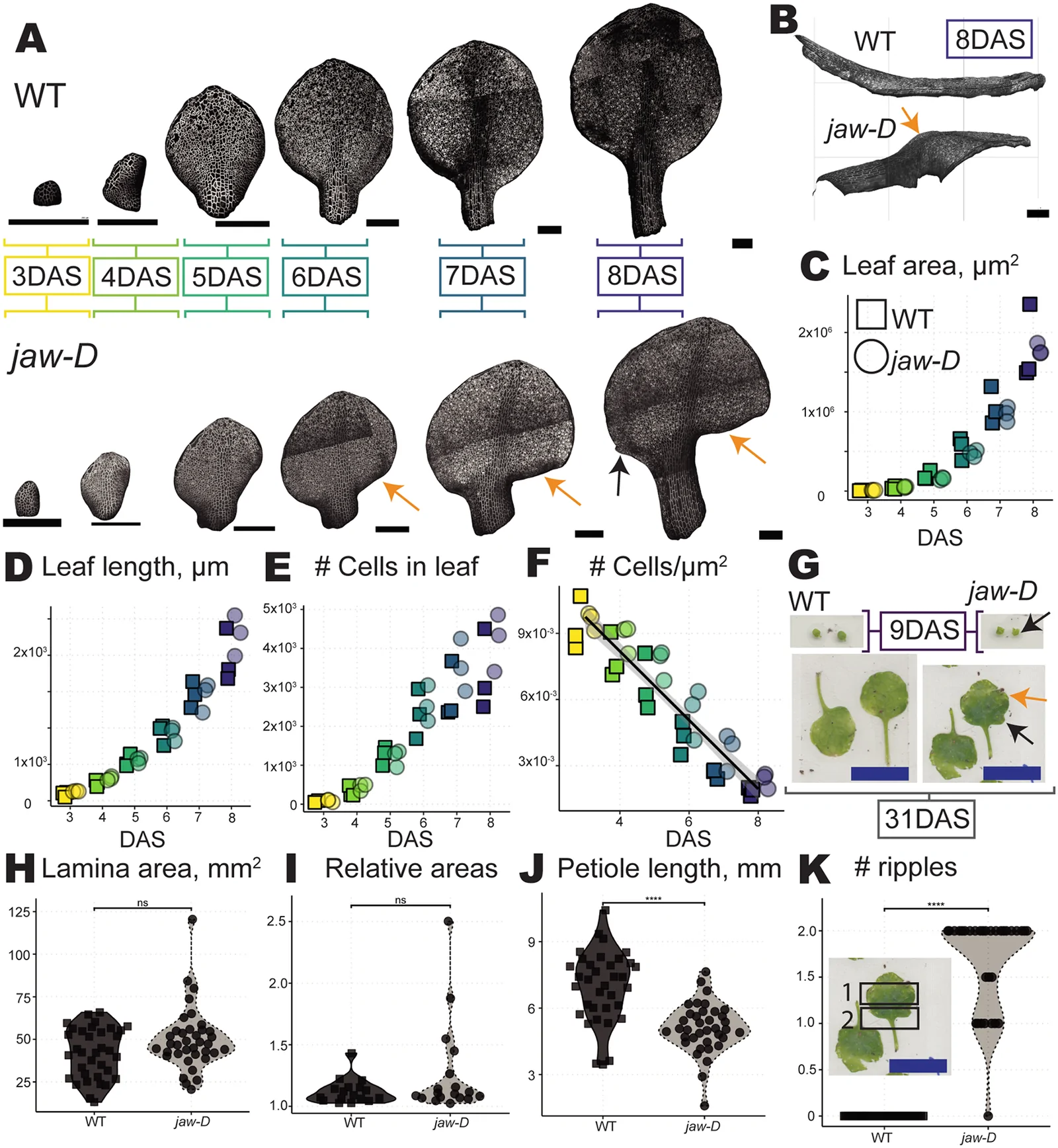
Features of *jaw-D* leaf form emerge soon after primordia initiation. **(A)** Fluorescent plasma membrane marker signal from confocal stack images of wild type (WT; top) and *jaw-D* (bottom) leaves from 3 DAS to 8 DAS are shown projected onto 2.5D surface meshes rendered in MorphoGraphX 2.0. Tissue differentiation in WT and its disruption in *jaw-D* can be seen starting at 5 DAS. Black arrows indicate the beginnings of leaf dimples and orange arrows indicate increased curvature in early *jaw-D* leaves that will become ripples at maturity. *n* = 3 leaves for each genotype. Black scale bars = 200 µm. **(B)** Side view of 8 DAS leaf meshes, with petiole on the left and tip on the right, show a global increase in leaf curvature in *jaw-D* (lower) vs. WT (upper). **(C, D)** Total segmented leaf area **(C)** and length **(D)** increase exponentially over the course of the live imaging experiment in WT and *jaw-D* plants. **(E)** The number of segmented cells in WT and *jaw-D* increases throughout the period imaged. **(F)** Cell density was determined as the number of cells per area segmented [cell density = # cells/sum(cell areas)] across the leaf. The density is mostly linear in *jaw-D* and WT. Squares indicate WT leaves, circles indicate *jaw-D* leaves. Colors indicate DAS. Means for all measures for each time point were not significantly different (paired Student *t* tests with Bonferroni correction). **(G)** Comparison of the first and second leaves of WT (left) and *jaw-D* (right) from 9 DAS (top) and mature leaves, at least 31 DAS (bottom). Blue scale bars indicate 10 mm. **(H)** Final lamina area for the first and second leaves of WT and *jaw-D*. WT leaf areas exhibit a broad and bimodal distribution, whereas *jaw-D* leaf areas center tightly around the median with more extreme values (*p* > 0.05, Wilcox test). **(I)** Relative area between the final sizes of the first and second leaves of the same plant in WT or *jaw-D. jaw-D* first and second leaves on the same plant have a greater spread of values and more extreme values (Feltz and Miller asymptotic test of CV *p* = 3.051466e-05). **(J)** Length of petioles in fully grown first and second leaves of WT and *jaw-D. jaw-D* petioles are shorter (*p* < .0001, Wilcox test). **(K)** Number of ripples in mature *jaw-D* plants (versus none in WT *p* < .0001, Wilcox test). Inset indicates identification of ripples in mature *jaw-D* plants. Color, shape and line type indicate genotype (solid, dark gray, squares = WT, dotted, light gray, circles = *jaw-D*). See S1–S6 Figs for three replicate wild type and *jaw-D* leaves showing the change in size, shape, and differentiation from 3 DAS to 8 DAS. See S7 Fig for further quantification of wild-type and *jaw-D* leaf growth. The data underlying this figure can be found at https://doi.org/10.17605/OSF.IO/D7X3Y, and the R code to create the graphs from the data is available at https://doi.org/10.17605/OSF.IO/YTVW9.

To understand how the three-dimensional form of the leaf arises from cellular growth patterns, we repeatedly imaged the same living wild-type and *jaw-D* mutant first leaves every day from 3 days after sowing (DAS) to 8 DAS (*n* = 3 leaves for each genotype; Figs 1A and S1–S6). The first two leaves develop simultaneously and are indistinguishable, so we consider them together as leaf 1 and 2. To increase the surface of the leaf that could be imaged, the cotyledons were dissected off [52]. We used confocal microscopy to image nearly the entire abaxial (bottom) epidermis of the leaf at cellular resolution so that we could relate cellular properties to organ-scale measurements. Then we used MorphoGraphX 2.0 image analysis software to represent the confocal stacks as two-dimensional mesh surfaces curved in three dimensions (2.5D) [53,54]. Cell boundaries were marked with a fluorescent plasma membrane marker. We segmented the epidermal cell signal projected on this mesh and tracked each of the cell lineages over time (S1–S4 Videos). This dataset serves as the basis for our further analysis of how cell growth and division give rise to organ shape (Dataset available at https://doi.org/10.17605/OSF.IO/D7X3Y).

To ensure that we had captured the relevant growth events that establish leaf flatness in wild type and curvature in *jaw-D*, we examined large-scale leaf development in our images during the 3–8 DAS that we imaged. Starting at 6 DAS, tissue curving became evident in *jaw-D* leaves, while wild-type leaves remained relatively flat (Fig 1A and 1B). This tissue curving continued throughout the experiment and was accompanied by ruffling along the margin at 8 DAS (Fig 1A, 1B, and 1G; S5 Video). In all samples, we observed rapid cellular and organ-wide morphology changes characteristic of early leaf growth (Figs 1A and S1–S6). Notably, at 4 DAS stomata began to initiate at the distal end of the leaf in both wild type and *jaw-D*, indicating that the basipetal maturation and differentiation gradient had initiated at the distal leaf tip. At 5 DAS, the petiole (stalk that attaches the leaf to the stem) initiated as a cylindrical fold in the proximal base of the leaf, differentiating it from the lamina (the flat leaf surface) in both wild-type and *jaw-D* leaves.

Organ-wide morphological features of the 3–8 DAS leaves, including area, length, width, cell number, and cell density, were not statistically different between wild-type and *jaw-D* (paired Student’s *t* tests with Bonferroni correction, Figs 1C–1F and S7A)*.* Therefore, we concluded that wild-type and *jaw-D* leaves could be considered at the same stage for each day in the experiment. However, the enhanced curvature of *jaw-D* prevented cell segmentation in some of the proximal leaf blade regions, so it is likely that proximal *jaw-D* cells are undercounted. Leaf area and leaf length continued to increase exponentially throughout the experiment (log(leaf area) ~ time, *R*^2^ = 0.93, *p* < 2.2e−16; log(leaf length) ~ time, *R*^2^ = 0.94, *p* < 2.2e−16, S7F and S7G Fig). Cell number also increased throughout the experiment (Fig 1E) [1]. Additionally, the density, the number of cells per area across the leaf, stayed mostly linear (cell density ~ time, *R*^2^ = 0.92, *p* < 2.2e−16; Fig 1F). Thus, with our imaging of 3–8 DAS, we captured both the generation of flatness/curvature and the basipetal cell division and growth gradient.

To relate the phenotypes that we captured early in leaf development to phenotypes of fully developed leaves, we scanned wild-type and *jaw-D* leaves 1 and 2 at maturity (at least 31 DAS). When we measured the mature first true leaves of wild-type and *jaw-D* plants, *jaw-D* leaves could not be flattened and therefore small tears at the margin had to be induced to measure leaf characteristics, as reported previously (Fig 1G–1K) [36]. By measuring paired leaves 1 and 2 from >15 plants each (wild-type *n* = 34 leaves, *jaw-D n* = 36 leaves), we observed that leaves undergo approximately a 400-fold increase in area from 8 DAS to maturity, yet the gross morphology is established by 8 DAS. Generally, the first two leaves of *jaw-D* form two ripples, and these ripples are established during the live imaging experiment window, while wild-type leaves never form ripples (Fig 1A, 1B, 1G, and 1K). Wild-type and *jaw-D* leaves have the same median size at maturity (Fig 1H, Wilcox test *p* > 0.05). Other measurements of organ size between wild type and *jaw-D* are likewise comparable (S7B–S7E Fig). We did find that the petiole of *jaw-D* is shorter than wild type (Fig 1J, Wilcox test *p* < .0001).

To test the robustness of organ size, we next asked whether leaves 1 and 2 are the same size (i.e., robust) in a single plant. We divided the area of the larger leaf from the pair by the area of the smaller leaf. If leaves are equal in size, the result is one. The median value was close to 1 and not statistically significantly different between wild-type and *jaw-D* plants (Fig 1I, 1.09 wild type versus *jaw-D* 1.10, Wilcox test *p* > 0.05), indicating in most plants the leaves were very close to the same size, and thus robust. However, *jaw-D* exhibited more outlier plants with extremely different leaf 1 and 2 sizes (statistically higher coefficient of variation Feltz and Miller Asymptotic test *p* = 3.05e−05), indicative of higher size variability induced by the *jaw-D* mutation.

We conclude that our live imaging of the early stages of leaf development (3–8 DAS) captures the initial development of organ shape at cellular resolution. We were curious how leaves with approximately the same starting and ending tissue material could have such distinct shapes, namely flattened in wild type and curved in *jaw-D*. In the next sections, we associate the spatial and temporal initiation of this curvature with cellular growth, division and differentiation patterns.

### While wild-type leaves progressively flatten, *jaw-D* leaves curve at the base

**Fig 2:**
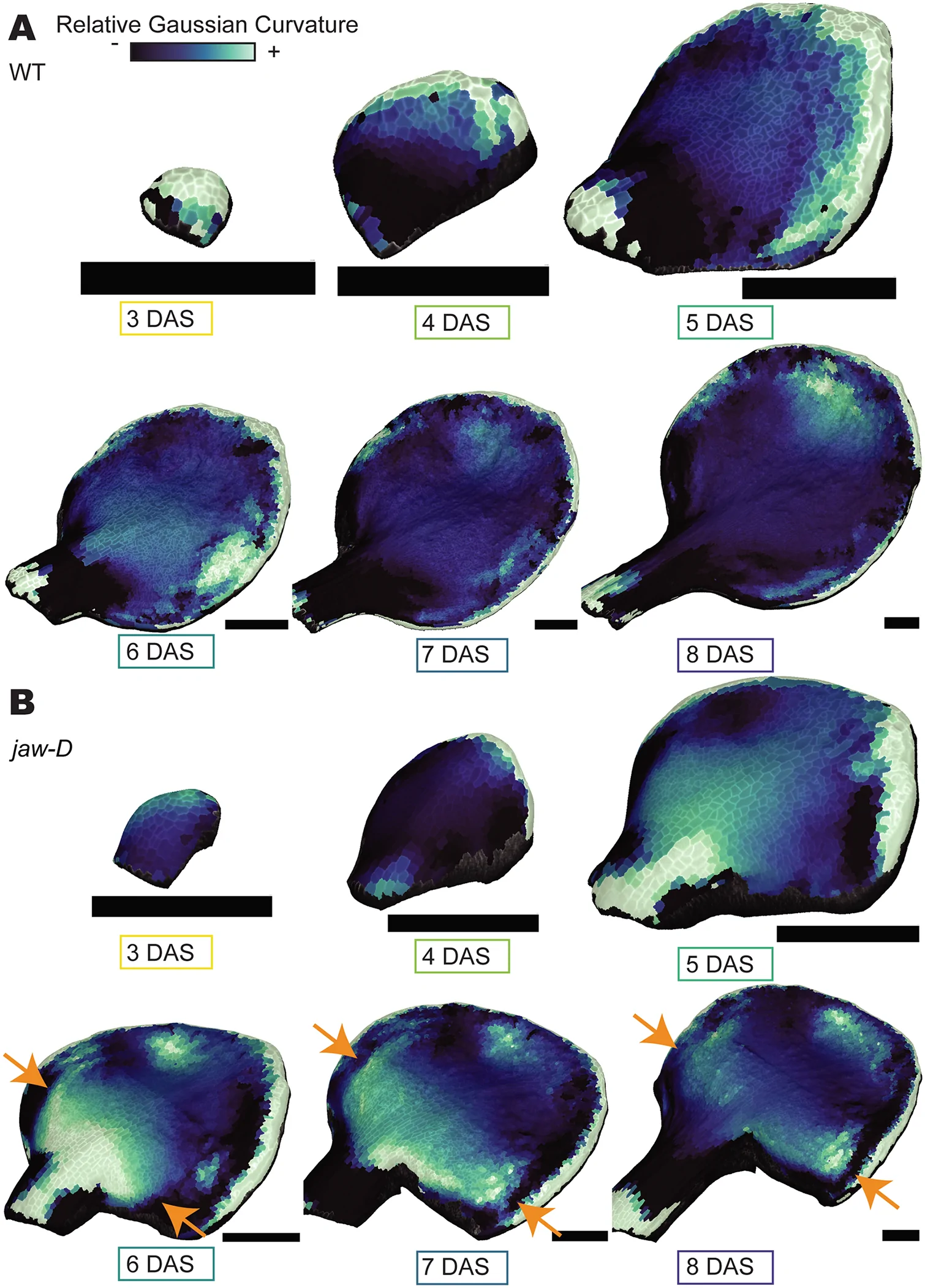
While WT leaves progressively flatten, *jaw-D* leaves curve at the base. **(A, B)** Gaussian curvature displayed as heatmaps on WT **(A)** and *jaw-D* **(B)** leaf meshes. Gaussian curvature represents both the magnitude and direction of curvature such that values further from zero represent greater curvature. Orange arrows indicate high positive curvature that develops at the base of *jaw-D* leaves. Black scale bars = 200 µm. These images are representative of three replicate live imaging series for each WT and *jaw-D*. Note that, as all leaves flatten, the Gaussian curvature changes dynamically over multiple orders of magnitude during the experiment. Further, the petiole and margin are sources of extreme outliers in curvature. Therefore, the scale for the heatmaps representing each time point has been calculated to encompass the 85% range of curvature values around the mean for that time point. See S8 Fig for quantification of curvature. The data underlying this figure can be found at https://doi.org/10.17605/OSF.IO/D7X3Y.

To quantify the magnitude and direction of tissue curvature, we calculated the Gaussian curvature for live-imaged wild-type and *jaw-D* samples (Figs 2 and S8). The sign of Gaussian curvature indicates the structure: a hill or valley (positive) or saddle (negative). Values of Gaussian curvature further from zero indicate greater curvature. At 6 DAS, *jaw-D* leaves establish a strong positive curvature across the proximal end of the leaf lamina as the whole leaf curves (Figs 2B and S8A). At early time points (3–5 DAS), wild-type leaves exhibit a range of curvature values (S8A Fig). At 6 DAS, while *jaw-D* leaves curve, wild-type curvature values along the length and width tighten around 0, as the leaf flattens (S8 Fig). We conclude that the entire *jaw-D* leaf curves, not just the margins, and that curvature is initiated at 6 DAS.

### Wild-type leaves exhibit a well-defined proximal–distal gradient of growth and division, which is lacking in *jaw-D*

**Fig 3:**
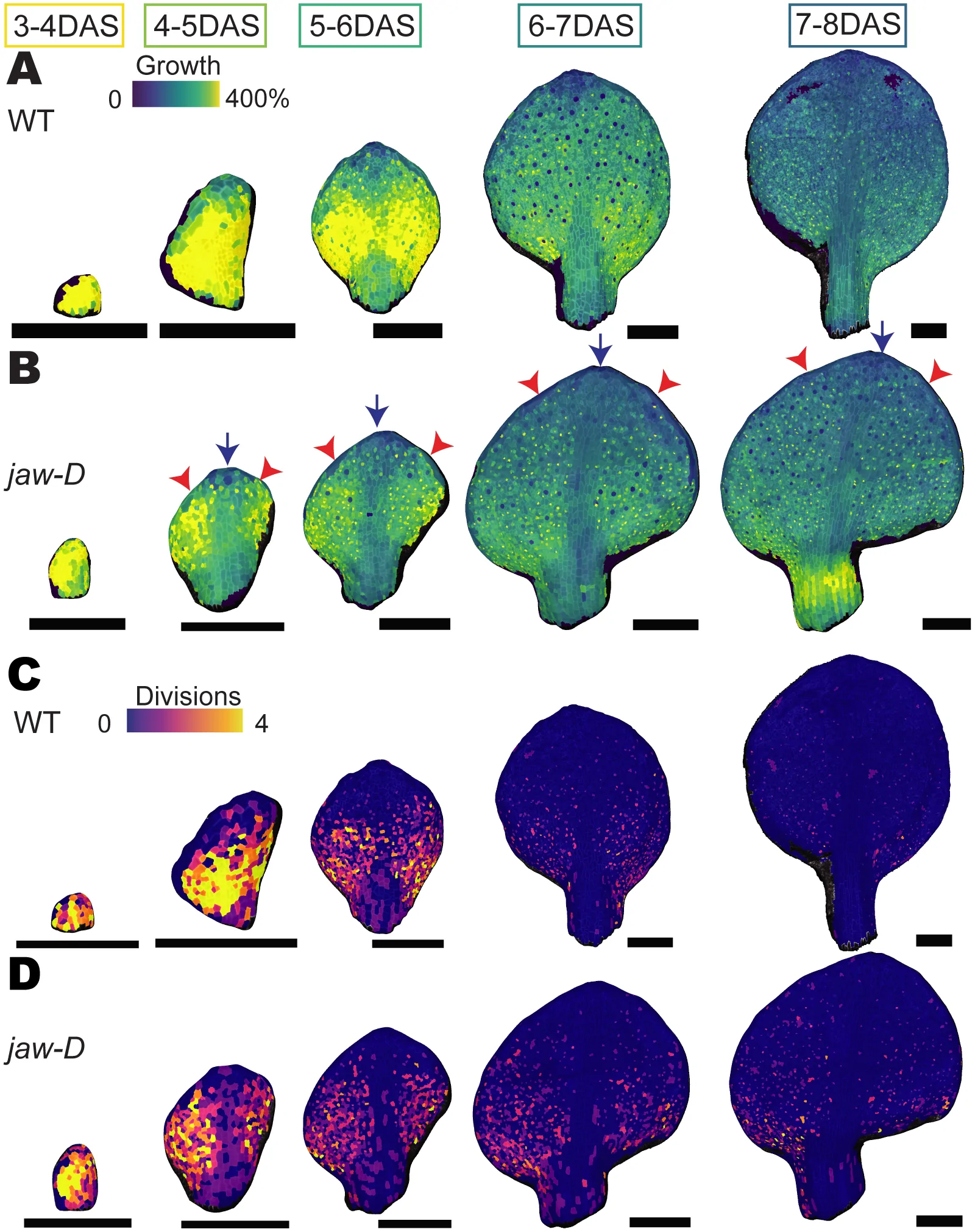
WT leaves exhibit a well-defined basipetal gradient of growth and division and uniform growth from midrib to margin, both lacking in *jaw-D.* **(A, B)** Heatmaps of cell areal growth (from 400% fast growth in yellow to 0% no growth in navy blue) in 24-hour imaging periods represented on sample WT (A) and *jaw-D* (B) leaf meshes on the earlier time step. Note the slowing of growth that initiates from the distal tip at 4–5 DAS and progresses toward the base in WT. This basipetal gradient is less obvious (somewhat flattened) in *jaw-D*. Note also that in WT the growth rate is the same from midrib to margin at a specific location on the proximal–distal axis, whereas in *jaw-D* the midrib consistently grows slower (blue arrows) than the leaf blade (red arrows). **(C, D)** Heatmaps of the number of cell divisions (from 4 in gold to 0 in purple) in 24-hour imaging periods represented on sample WT (C) and *jaw-D* (D) leaf meshes on the earlier time step. Note the slowing of division that initiates from the distal tip at 4–5 DAS and progresses toward the base in WT. This basipetal gradient is less obvious (somewhat flattened) in *jaw-D*. Black scale bars = 200 µm. See S9 Fig for spatiotemporal quantification of growth in all three biological replicates. The data underlying this figure can be found at https://doi.org/10.17605/OSF.IO/D7X3Y.

To investigate the relationship of cells to organ-level curvature, we analyzed the patterns of cellular growth and division in the leaf. From our segmented and lineage-tracked cells, we calculated the relative growth rate (area of the cell/cell lineage at the later time point as a percent of the cell area at the earlier time point) and number of divisions undergone by each cell at each time point in the live imaging experiment (Fig 3A–3D). To quantify the relationship between cell division and growth in tandem, we represent each of these values for each cell in its normalized proximal–distal and mediolateral position (S9 Fig).

Wild-type leaves have previously been shown to have a basipetal growth gradient, in which slowing growth and cessation of cell division initiate in the tip and progress downward. Thus, at many developmental stages, wild-type leaves exhibit fast growth in the proximal base and slow growth in the distal tip. Given that TCPs promote slowing growth and cessation of cell division to create the basipetal growth gradient in wild-type leaves, we expected the basipetal growth gradient to be disrupted in *jaw-D* because overexpression of the *miR319* inhibits TCPs [36,37,39,40]. As anticipated, the basipetal growth gradient that is prominent in wild type is perturbed in *jaw-D.* In both wild type and *jaw-D* over 3–4 DAS, the whole leaf primordium grows and divides rapidly (Fig 3). At 4–5 DAS in both wild type and *jaw-D*, cells in the distal tip slow their growth and stop dividing, initiating the basipetal growth gradient (Fig 3). In wild type, slowing growth and cessation of cell division proceed down the leaf blade over 5–8 DAS (Figs 3A, 3C, and S9). In contrast, in *jaw-D*, a moderate growth and division rate extends across the leaf blade (Figs 3B, 3D, and S9). All three leaf replicates for each genotype exhibit these respective growth patterns (S9 Fig). Thus, the basipetal growth gradient initiates at the distal tip of *jaw-D* leaves, but does not properly progress down the leaf over time as it does in wild type.

**Fig 4:**
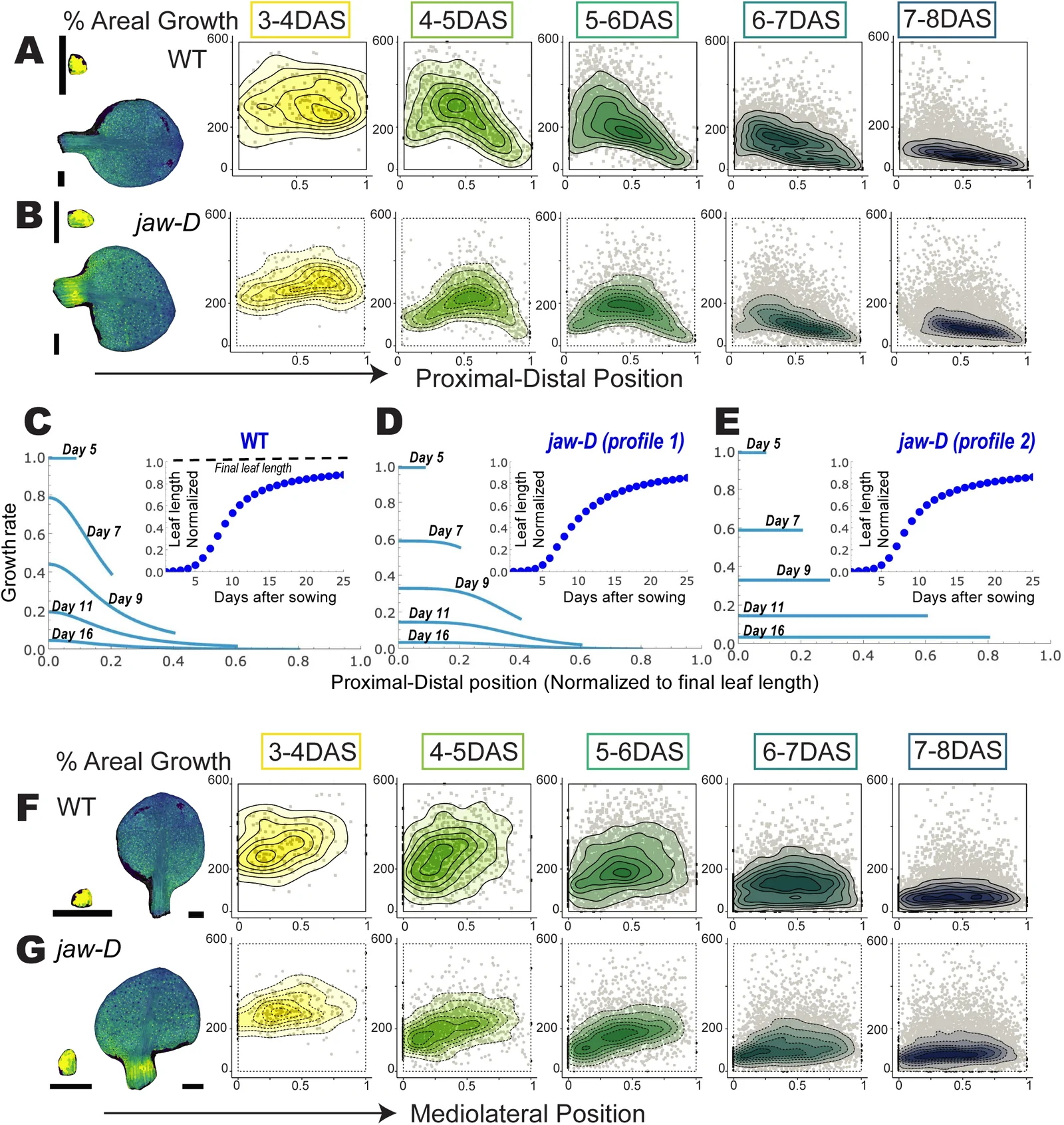
Quantification of disruption in the basipetal growth gradient and midrib-to-margin growth in *jaw-D* vs. wild type. **(A, B)** Density plots of each cell’s percent cell area increase in 24 hours graphed according to cell position along the proximal–distal axis (normalized so the tip is 1 and base is 0) to examine the basipetal growth gradient. On days 4–5 and 5–6, there is a pronounced slope with slower growth (blue arrow) in the tip and faster growth in the base (red arrow) of wild-type leaves, showing the basipetal growth gradient. At the same time points, the cell area increase in *jaw-D* is more uniform across the length of the leaf with the highest areal growth percentages in the center (red arrow) compared to both ends (blue arrows). The images of the areal growth heatmaps (reproduced from Fig 3) illustrate the alignment of the proximal–distal axis in the graphs. Black scale bars = 200 µm. **(C–E)** The modeled growth profile along the leaf proximal–distal axis, and its corresponding leaf length dynamics for WT **(C)**, and for two different *jaw-D* growth profiles **(D, E)** that result in similar leaf length across days as the WT. **(F, G)** Density plots of each cell’s percent cell area increase in 24 hours graphed according to cell position along the mediolateral axis (normalized so the margin is 1 and midrib is 0) to examine the uniformity of growth in the mediolateral direction. Note that from 4–5 and 5–6 DAS the growth near the midrib is lower (blue arrows) than in the blade in *jaw-D*. The images of the areal growth heatmaps (reproduced from Fig 3) illustrate the alignment of the mediolateral axis in the graphs. Black scale bars = 200 µm. See S10 Fig for quantification of cell divisions. The data underlying this figure can be found at https://doi.org/10.17605/OSF.IO/D7X3Y and the R code to create the graphs from the data and the code for the model in C–E is available at https://doi.org/10.17605/OSF.IO/YTVW9.

To further quantify the defects in the basipetal growth gradient in *jaw-D*, we examined the level of daily areal growth and divisions of cells relative to their position along the proximal–distal axis of the leaf. Wild-type leaves show the classic basipetal growth gradient with low growth at the distal tip and high growth rates near the proximal base from 4–7 DAS (Fig 4A). We found a striking shift in the distribution of areal growth in *jaw-D* leaves along the proximal–distal axis, with growth occurring at a more constant rate all the way along the proximal–distal axis. The slight maximum in growth in *jaw-D* occurs near the middle of the leaf throughout 4–6 DAS when the change in curvature initiates in *jaw-D* (Fig 4B). Cell divisions showed similar trends (S10A and S10B Fig) with the *jaw-D* distribution centered near the middle half and the wild-type distribution decreasing from a maximum in the proximal quarter of the leaf. Thus, the basipetal growth and division gradient of wild type is spatially flattened at each time point in the *jaw-D* leaf. This suppression of the basipetal growth gradient in *jaw-D* is consistent with the current understanding of disruption of the *miR319 TCP* network in *jaw-D*. TCP transcription factors promote maturation from the tip downward [36,37,39,40]. Ectopic expression of *miR319* in *jaw-D* leads to expanded repression of *TCP* expression and consequently is expected to suppress the basipetal growth gradient as observed in our live imaging analysis.

We were struck by the observation that the *jaw-D* leaf does not become longer than wild type (Fig 1D) despite having an expanded region of fast growth and division (Fig 3). To explore how contrasting spatial growth profiles for wild-type and *jaw-D* genotypes can lead to overall similar leaf length dynamics, we created a simple one-dimensional model to capture the length of the leaf over time. In the model, we assume growth is initially exponential in both wild type and *jaw-D*. As the leaf dynamics exit this initial exponential phase, for wild type, we implement a basipetal growth gradient curve (Fig 4C). We also assume that the growth at the leaf base decreases over time; this decrease is essential to eventually stop all growth as the leaf length approaches its steady-state value (Fig 4C). For *jaw-D,* first we decreased the steepness of the basipetal growth gradient. Consistent with our data, the model predicts similar leaf lengths can be maintained between wild type and *jaw-D* despite these different growth profiles (Fig 4D). We then further simplified the pattern as having no basipetal gradient and implemented a constant moderate growth rate along the leaf length (Fig 4E). Even this very simple model produced similar leaf lengths as wild type (Fig 4E). Based on the model, we conclude that different proximal–distal growth patterns can yield similar leaf lengths. This model hints that proximal–distal growth defects alone cannot explain the curvature phenotype. Thus, a spatial flattening of the basipetal growth gradient is one of the major defects in *jaw-D* mutants, but it was not yet clear how alterations in *jaw-D* cellular growth affect curvature.

### In *jaw-D* leaves, the midrib grows slower than the leaf blade, whereas wild-type growth is more even across the mediolateral axis

To look for further defects in the cellular growth of *jaw-D* that could give rise to changes in curvature, we also examined growth in the mediolateral (middle to edge) leaf axis. In previous publications, leaf ruffling has been attributed to overgrowth along the leaf margins [7,35]. We did not observe any obvious overgrowth or excessive division in the edges of *jaw-D* leaves beyond that in wild type in the first leaves (Fig 3). It is possible that overgrowth along the margins occurs in *jaw-D* leaves that develop later on the plant. We note that our live imaging captured the first of two ripples that will be formed in the first leaves of *jaw-D* (Fig 1B and 1G). We also note that we use the term ripple, because the phenotype is not just at the margin, but curvature that extends across the whole leaf. Since curvature is affected across the *jaw-D* leaf, not just the margins, we looked more broadly for defects in growth in the mediolateral direction. The *jaw-D* midrib (middle of the leaf overlying the central vascular bundle) grows more slowly than the leaf blade from 4 to 8 DAS (Fig 3B). In contrast, the wild-type midrib and blade have nearly equivalent growth rates along the same latitudinal level (Fig 3A). Further quantifying growth rates along the mediolateral axis confirmed slower growth near the midrib and faster growth throughout the blade in *jaw-D*, whereas growth rates were more broadly and evenly distributed in wild type (Fig 4F and 4G). Cell divisions largely cease at 5–6 DAS and are less distinguishable mediolaterally between wild type and *jaw-D* (S10C and S10D Fig). So, we reasoned that growth, rather than divisions, was a major driver in creating the curvature differences in *jaw-D* leaves. Our data is consistent with the hypothesis that differences in growth rate between the midrib and the leaf blade in the mediolateral axis create mechanical conflicts that cause the rippling of the whole *jaw-D* leaf. In contrast, the relatively even growth rate in the midrib and blade in wild type would not cause conflicts so the leaf can retain flatness.

In summary, our growth analysis revealed two major defects in *jaw-D* growth relative to wild type. First, in the proximal–distal direction, the basipetal growth gradient is spatially flattened in *jaw-D* compared to wild type. Second, in the mediolateral direction, the midrib growth is slower than the rest of the leaf blade in *jaw-D*, whereas the midrib grows as fast as the blade in wild type. Therefore, we examined the growth and development patterns of the leaves along these two axes in more detail to understand how cellular growth gives rise to curvature in *jaw-D* and flatness in wild type.

### Analysis of growth rate variability confirms the two major defects in *jaw-D* growth patterns: Flattened basipetal growth gradient and slow growth at the midrib

**Fig 5:**
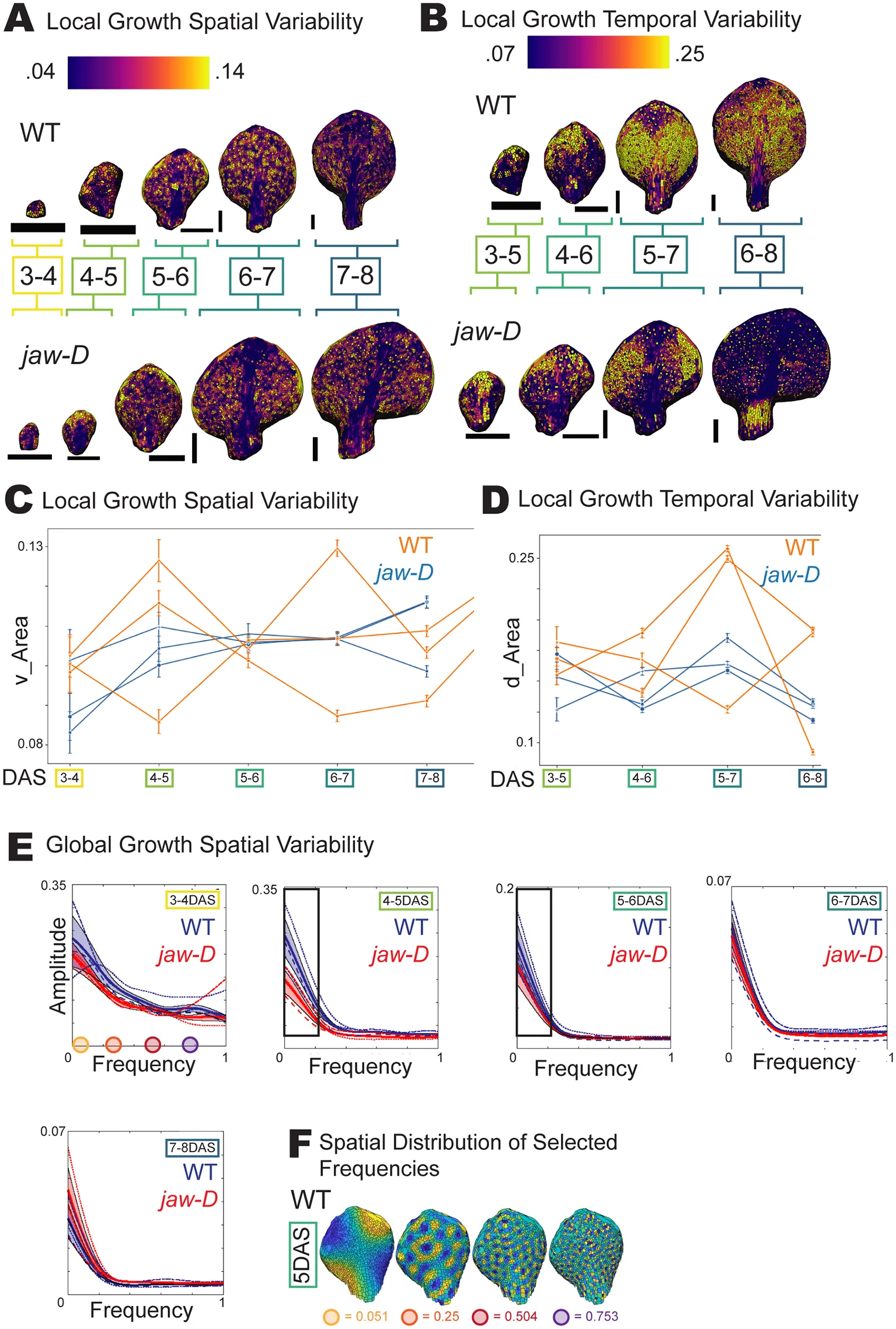
Global growth variability dominates in WT leaves. **(A)** Heatmaps of local spatial growth variability over 24-hour windows represented on sample WT (top) and *jaw-D* (bottom) leaf meshes on the earlier time step. Gold indicates high spatial variability and purple low spatial variability. See Materials and methods for spatial variability calculation. **(B)** Heatmaps of local temporal growth variability in 48-hour windows, represented on sample WT (top) and *jaw-D* (bottom) leaf meshes on the middle time step. Gold indicates high temporal variability and purple low temporal variability. See methods for calculation of temporal variability. Black scale bars = 200 µm. **(C, D)** Quantification of the mean (C) local growth spatial variability or (D) local growth temporal variability for time windows as in **(A-B)**. Individual lines connect mean values for each of three WT (orange) or *jaw-D* (blue) replicates. Error bars indicate standard error of the mean. WT and *jaw-D* local spatial and temporal growth variability are largely indistinguishable, though *jaw-D* replicates are generally tighter around the mean. **(E)** Global spatial variability of areal growth for 24-hour windows determined through cellular Fourier analysis. High amplitudes at lower frequencies represent higher variability at larger length scales. Lines represent each of three WT (blue) or *jaw-D* (red) replicates with root mean squared error shaded. At 4–5 DAS and 5–6 DAS WT leaves exhibit much higher variability in growth across the organ than *jaw-D* (boxed). **(F)** Example of spatial range for different frequencies displayed on 5DAS WT leaf. Colored dots indicate the frequency value on the x-axis of (E) panel 1. See S11 Fig for spatiotemporal variability analysis excluding stomata. The data and analysis code underlying this figure can be found at https://doi.org/10.17605/OSF.IO/YTVW9.

To further characterize the cellular growth patterns, we quantified the local variability in growth rates, i.e., how different is the growth rate of a cell from its neighbors and how much does the growth rate of an individual cell change between one time point and the next [55]. In both local spatial and temporal variability, *jaw-D* shows a prominent reduction in cell growth variability at the midrib between 5 and 8 DAS (Fig 5A and 5B). The lower variability in midrib cell growth confirms the persistent slower growth rate of the midrib relative to the more dynamic growth of the leaf blade. In contrast, the midrib is not as distinct in wild type because the midrib cell growth is more equivalent to that in the leaf blade (Fig 5A and 5B). If we quantify the overall local spatial and temporal variability by averaging cells throughout the leaf, we found that wild type and *jaw-D* are largely indistinguishable (Fig 5C and 5D), indicating that the localized pattern, not the global pattern is important in the case of mediolateral growth. Stomata have recently been suggested as the source of most growth heterogeneity in tissues [56], which is also evident in our heatmaps (Fig 5A and 5B). However, our analysis was largely unaffected when stomata were excluded (S11 Fig).

To examine larger patterns of growth change across the organ, we next applied a cellular Fourier analysis, which quantifies the heterogeneity in growth of the tissue across all length scales [57,58]. We found that there was less large-scale variation in growth rate across the organ in *jaw-D* than wild type (Fig 5E and 5F). Our cellular Fourier analysis also emphasized that the globally heterogeneous growth present in wild type and reduced in *jaw-D* is prevalent at 4–6 DAS, when the curvature in *jaw-D* is established (Fig 5E black boxes)*.* At first glance, this reduced heterogeneity in *jaw-D* may seem paradoxical, since curvature is often thought to arise from conflicts in differential growth. However, growth variations do not inherently generate mechanical incompatibility, as the emergence of curvature depends on the orientation and spatial structure of growth gradients, not simply on the magnitude of growth variability. In *jaw-D*, the basipetal growth gradient is flattened while the mediolateral growth gradient is enhanced. Our Fourier analysis suggests that the flattening of the basipetal gradient outweighs the increase in mediolateral variation, with distinct mechanical consequences: the strong basipetal gradient in wild type can be accommodated without conflict, whereas the enhanced mediolateral gradient in *jaw-D* may introduce localized conflicts that produce curvature. We therefore investigate further how the relationship between growth gradients and curvature gives rise to the observed phenotype.

### Clonal analysis reveals contribution of progenitor cells to the leaf

**Fig 6:**
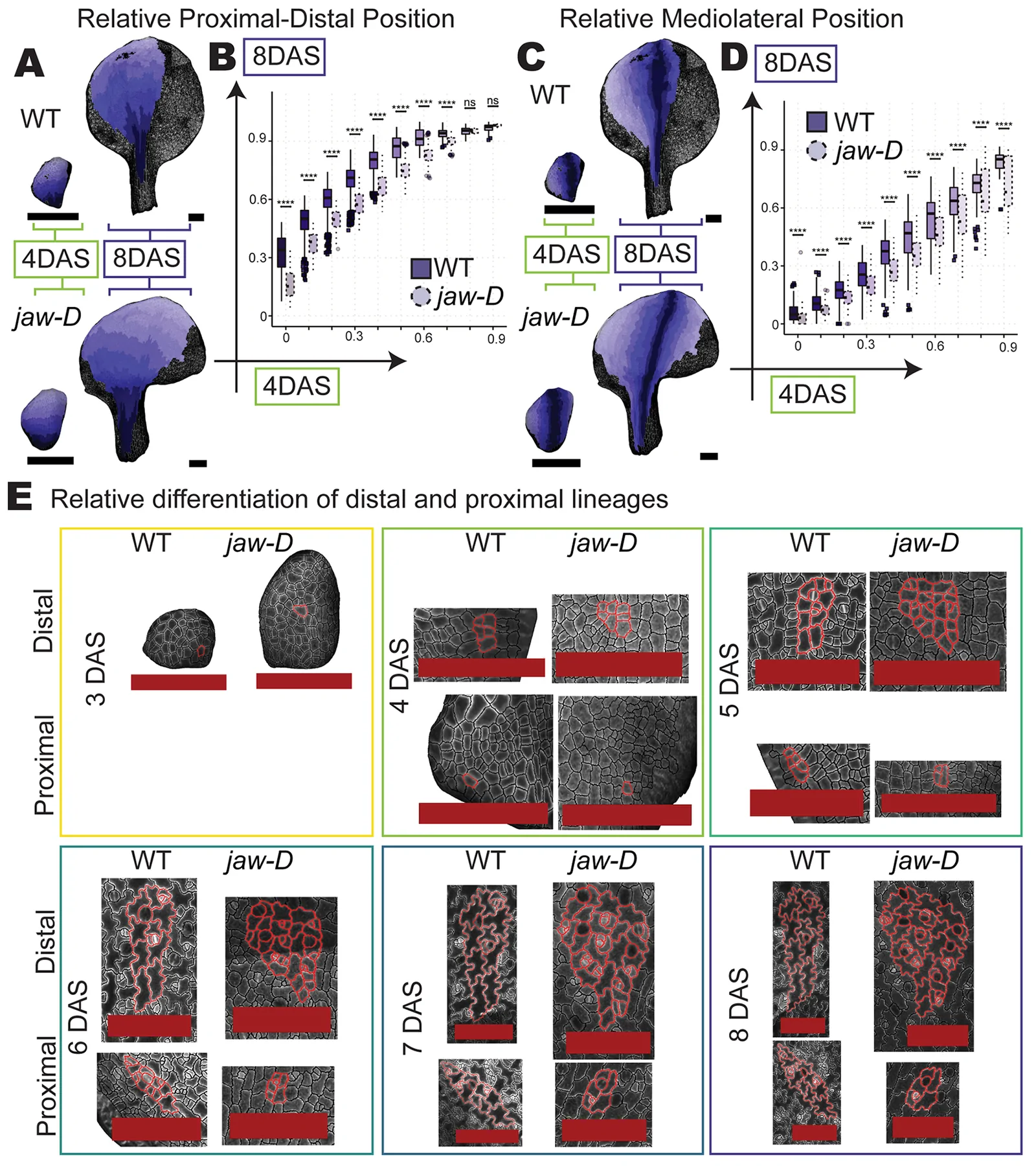
Cell differentiation quickly propagates from the distal tip along the leaf length in WT, unlike *jaw-D.* **(A, C)** Clonal analysis of WT (top) and *jaw-D* (bottom) cells mapped from 4 DAS to 8 DAS on sample leaf meshes. Clonal sectors (shades of purple) were binned evenly at 4 DAS into 10 proximal–distal (A) or mediolateral (C) sectors. Uncolored cells could not be tracked between all time points. **(A)** Darker purple WT bins end up larger than lighter bins indicative of expanded contribution of proximal growth and divisions to the overall leaf vs. a more even distribution of colors in *jaw-D.* **(C)** Lighter purple bins are expanded in *jaw-D* indicating a lateral shift of sector contributions. Black scale bars = 200 µm. **(B, D)** Quantification of proximal–distal (B) or mediolateral (D) sector relationships for 4 DAS sector bins mapped to 8 DAS positions for all three replicates of WT (left, dark shading, solid lines) and *jaw-D* (right, light shading, dotted lines) whole leaves. Boxes indicate the 50% interquartile range, middle bar represents median, whiskers represent outliers and squares (WT) or circles (*jaw-D)* represent extreme values. Nearly all 4 DAS sector bins have different mapping to 8 DAS positions between WT and *jaw-D* representing more prominent contributions of WT proximal and medial regions than disbursed and lateral contributions in *jaw-D* (*p* < .0001, paired Student *t* test and Bonferroni correction)*.* **(E)** Tracing the same proximal (bottom) and distal (top) cell lineages (red outlines) for the entire experiment for WT (left) or *jaw-D* (right) sample leaves. WT cells exhibit greater differentiation into lobed pavement cells starting at 5 DAS. Red scale bars = 100 µm. See S12 Fig for further clonal analysis. The data underlying this figure can be found at https://doi.org/10.17605/OSF.IO/D7X3Y, and the R code to create the graphs from the data is available at https://doi.org/10.17605/OSF.IO/YTVW9.

To understand the extent to which each progenitor cell at 4 DAS contributes to the final leaf at 8 DAS, we analyzed the clonal sectors: the set of daughter cells at 8 DAS derived from a single progenitor cell at 4 DAS based on our lineage tracking data (S1–S4 Videos). Given the basipetal growth gradient in wild type, we expected cells near the distal tip of the 4 DAS leaf to differentiate early and contribute relatively little to the leaf blade, whereas cells at the proximal base would be expected to differentiate later and contribute a much larger fraction of the leaf. Progenitor cells at 4DAS were assigned into 10 even proximal–distal bins based on their position (Fig 6A and 6B). Then their contributions to the final leaf at 8 DAS were compared. As expected, cells from the wild-type proximal regions at 4 DAS contributed more to 8 DAS leaves in wild type (large region of the leaf filled with dark purple in Fig 6A) than cells from the distal tip (very small region of light purple in Fig 6A), consistent with the basipetal growth gradient. In contrast, in *jaw-D*, there was a more even contribution of progenitor cells along the proximal–distal axis to the final leaf (more equal stripes of each purple hue in Fig 6A). We further quantified this difference by comparing the relative location of the cell along the proximal–distal axis of the leaf at 4 DAS (i.e., 1.0 is the distal tip and 0.9 is 90% of the distance along the leaf) with the relative location of the clone at 8 DAS (Fig 6B). The most proximal cells at 4 DAS contribute to about 30% of the wild-type leaf whereas only about 15% of the *jaw-D* leaf (Fig 6B). Our results are consistent with the flattening of the basipetal growth gradient in *jaw-D* such that most regions of the leaf give rise to more equal amounts of leaf tissue. On the other hand, the most distal two tenths of both wild-type and *jaw-D* leaves had the same mean position from 4 DAS to 8 DAS, suggesting that the tip of the leaf differentiates close to 4 DAS and may be unperturbed in *jaw-D.* We similarly examined the contributions of cells along the mediolateral axis of the leaf and found that j*aw-D* showed a slight shift in the contribution of more lateral cells to the final leaf width (Fig 6C and 6D), which is consistent with the slow growing midrib region. Note that we also performed this analysis from 3 DAS to 8 DAS, but so much of the final leaf is contributed from the base in wild type that only about half of the leaf could be back-tracked (S12 Fig). The proximal–distal patterns still largely held; however, the mediolateral differences were much less pronounced.

### Proximal–distal tissue differentiation corresponds with the basipetal gradient of growth in wild type, differentiation is correspondingly slowed in *jaw-D*

To understand how these sectoral patterns relate to cell differentiation, we used cell lobing as a measure of the differentiation of pavement cells [59,60]. We chose two lineages with approximately equal beginning and ending positions between wild type and *jaw-D* to track over the entire experiment, a distal lineage that emerged at 3 DAS and a more proximal lineage that emerged at 4 DAS (Fig 6E). In wild-type leaves, pavement cell lobing increased starting at 5 DAS in the distal lineage and 6 DAS in the proximal lineage. Lobing in the *jaw-D* lineage began at 6 DAS, never became as elaborate as wild-type lobing, and further, was more similar between distal and proximal lineages. Our results in these lineages suggest delayed differentiation of pavement cells in *jaw-D* mutants.

**Fig 7:**
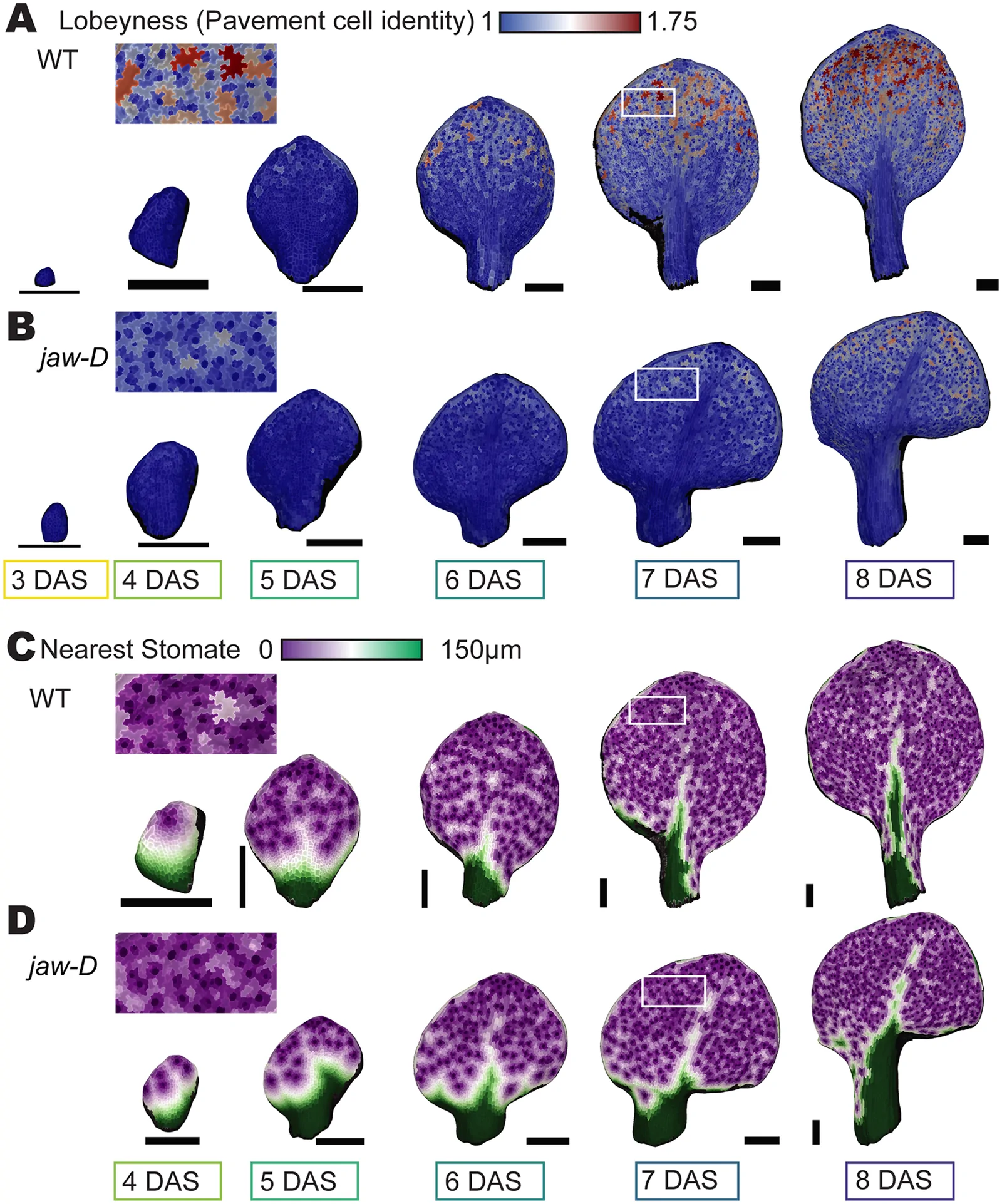
Proximal–distal tissue differentiation corresponds with the proximal–distal gradient of growth in WT, both disrupted in *jaw-D.* **(A, B)** Cell lobeyness (the perimeter of a cell divided by its convex hull, a measure of pavement cell differentiation). Represented as heatmaps on sample WT (A) or *jaw-D* (B) leaf meshes for each day of the imaging experiment. Black scale bars = 200 µm. Insets show magnified lobing from the 7 DAS leaf (white boxes). **(C, D)** Stomatal spacing (the distance in µm to the nearest stomate from any pavement cell, the inverse of stomatal density) represented as heatmaps on sample WT (C) or *jaw-D* (D) meshes for each day of the imaging experiment. Insets show magnified stomatal differentiation from the 7 DAS leaf (white boxes). See S13 and S14 Fig for further quantification. The data underlying this figure can be found at https://doi.org/10.17605/OSF.IO/D7X3Y.

The differences in pavement cell lobing observed in single lineages motivated us to measure global changes in the distribution of cellular differentiation relative to the cell growth patterns we measured in wild type versus *jaw-D.* We measured pavement cell lobing and stomatal characteristics to understand how two different types of differentiation proceed in the cells of wild-type and *jaw-D* leaves (Figs 7, S13, and S14). The lobeyness measure in MorphoGraphX allows us to ascertain the level of lobing present in segmented cells throughout the epidermis of a given leaf. We used this measure to ask how patterns of differentiation present along the proximal–distal and mediolateral axes change with leaf development. We found that in wild-type leaves, lobing begins to increase in the distal quarter of the leaf at about 5 DAS (Figs 7A and S13A). Over subsequent days, the lobeyness of many of these distal cells continues to increase and lobeyness initiation propagates from tip to base (Figs 7A and S13A). In contrast, *jaw-D* leaf cells initiate lobing 6 DAS or later, to a lesser extent and more evenly along the length of the leaf, suggesting differentiation is delayed and reduced in *jaw-D*, consistent with the spatially flattened basipetal growth gradient (Figs 7B and S13B)*.* Along the mediolateral axis, the differences in absolute magnitude of cell lobing are especially clear between wild-type and *jaw-D* samples (S13C and S13D Fig). This suggests that important differentiation signals at 5 DAS that are perceived in wild type, perhaps to reinforce and structure the growth directions, are lacking in *jaw-D.*

Stomata also initiate as leaf tissue matures, so we investigated whether stomatal differentiation was also impaired in *jaw-D.* In contrast to pavement cell lobing, we did not see such stark differences between wild type and *jaw-D* in stomatal density (Fig 7C and 7D). We measured stomatal density by labeling each stomate (labeled as one cell) in each leaf and then calculated the distance from any pavement cell to the nearest stomate. At 4 DAS, the spacing curve is nearly the same between wild type and *jaw-D* as the first stomata are initiated at the tip (S14 Fig). There is a slight lag in the flattening of this curve in *jaw-D* as stomatal initiation progresses down the growing lamina at 5 and 6 DAS. Yet, by 7 and 8 DAS both wild type and *jaw-D* have evenly spaced stomata (S14 Fig). We hypothesize this indicates the independence of the stomatal patterning process from tissue and organ-wide shape regulation.

Thus, the defective basipetal slowing of growth and cessation of cell division in the *jaw-D* mutant is accompanied by delayed and reduced basipetal progression of pavement cell differentiation. In contrast, stomatal patterning appears to be somewhat independent of this wave given that it progresses fairly normally in the *jaw-D* mutant.

### Longitudinal growth is coordinated across the width of the leaf blade to prevent growth conflicts, allowing flat development of the blade

**Fig 8:**
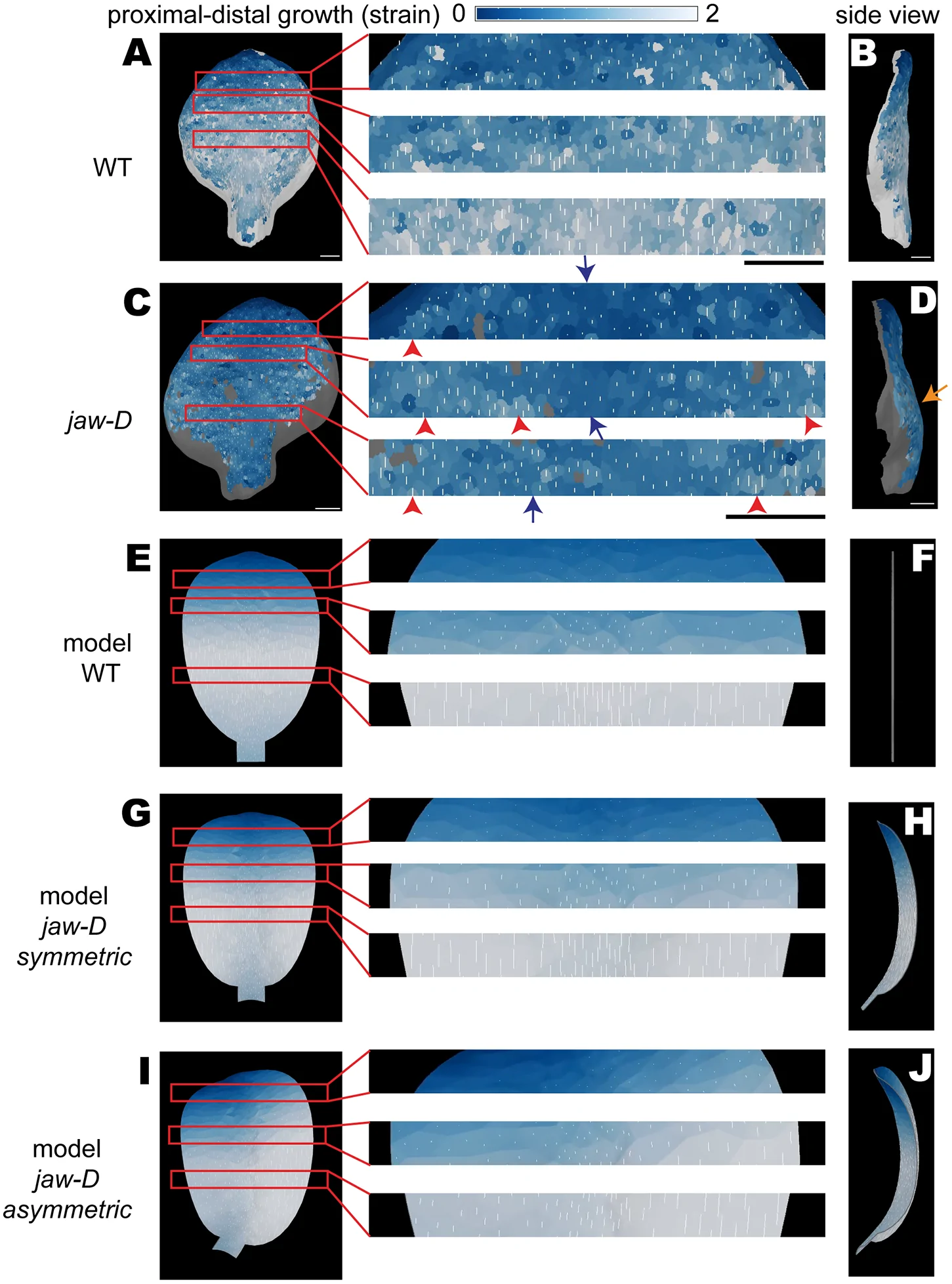
Coordination of growth rate across the width of the leaf blade prevents growth conflicts in wild type, keeping the leaf flat. **(A–D)** Heatmap of cell growth along the proximal–distal axis of the leaf over 24 hours from 5 to 6 DAS in WT (A-B) and *jaw-D* **(C, D)**. High proximal–distal strain (stretch −1) is colored white (2) and no extension is colored blue (0). The magnitude of growth along this proximal–distal axis is also represented by the length of the white line drawn in each cell. This stage of leaf growth was chosen for analysis because it immediately precedes the generation of high amounts of curvature in the *jaw-D* leaf. Three regions are magnified to make the cellular growth rates visible across the medial–lateral axis of the leaf. Note that the proximal–distal growth rates at each mediolateral region of the leaf are similar (allowing for typical growth variability) all the way across the leaf in WT, but not in *jaw-D* where coordination of the proximal–distal growth is disrupted and there are patches of faster (red arrows) and slower (blue arrows) growing cells. Note also the asymmetry between different sides of the *jaw-D* leaf. **(B, D)** A 90° rotation of the same leaf (side view), showing the flatness or curvature (orange arrow) of the leaf blade. Scale bars = 100 µm. **(E–J)** Finite element model simulation of leaf growth in which the proximal–distal growth is displayed. **(E, F)** When proximal–distal growth rates are equal across the width of the leaf, the leaf grows to a flat shape, similar to wild-type leaves. **(G, H)** When the growth of the midrib is less than the blades, a growth conflict causes the leaf to curve similar to *jaw-D* leaves. **(I, J)** When the growth of the midrib is lower than the blades and the growth of the blades are asymmetric, the leaf also curves in asymmetric shapes similar to some *jaw-D* leaves. See S15 Fig for mediolateral growth and S6–S7 Videos. The data underlying this figure can be found at https://doi.org/10.17605/OSF.IO/D7X3Y and code for the model is available at https://doi.org/10.17605/OSF.IO/YTVW9.

Cells within the leaf that grow at different rates create growth conflicts, which lead to out-of-plane bending and curvature [19,23]. To assess the directional growth conflicts in wild-type and *jaw-D* growing leaves, we examined the cell growth rates along the proximal–distal direction and mediolateral direction separately. We first examined proximal–distal growth at 5–6 DAS when the *jaw-D* leaf starts to curve. Given the basipetal growth gradient in wild type, we expected cells at the tip of the leaf to have a slower growth rate in the proximal–distal axis than cells near the base of the leaf, which was confirmed in our measurements (Fig 8A). In wild type, we further see that at each height (point along the proximal–distal axis), the growth rates are fairly uniform across the width (mediolateral axis) of the leaf (Fig 8A). We reasoned that the uniformity across the width would mean that this section of leaf would extend at the same rate, and therefore, would not create a growth conflict, allowing the leaf to remain flat. Examining the leaf from the edge confirmed that the wild-type leaf is flat (Fig 8B). Performing the same analysis of proximal–distal growth rates in *jaw-D* revealed regions of faster and slower growth across the width of the leaf at each height (Fig 8C). Particularly near the center of the leaf, the proximal–distal growth rates tended to be lower (Fig 8C), in keeping with the slow cell area growth rates observed in the midrib (Fig 3B). The *jaw-D* leaf starts to curve at the same time these uneven growth rates occur (Fig 8D).

We used finite element modeling to test how uniform and nonuniform patterns of proximal–distal growth affect the curvature of the leaf. We first created a model with a basipetal growth gradient in which the proximal–distal growth rate was uniform across the width of the leaf, representing wild type (Fig 8E and S6 Video). In the simulation of this model, each region of the leaf grew different amounts, but since the amount was equal across the width of the leaf, it did not create any growth conflict and the leaf remained perfectly flat, similar to wild type (Fig 8F). In contrast, when we altered the proximal–distal growth rate, so that it was non-uniform across the leaf, growth conflicts were created and the leaf curved similar to the *jaw-D* leaf (Fig 8G and 8H; S7 Video). Unlike the model, the real *jaw-D* leaf was grown between the mechanical constraints of a cover slip and the agar growth media, which is likely to cause the flattening of the tip of the leaf. We observed that *jaw-D* leaves that were not constrained in this way tended to roll back on themselves, preventing imaging, but consistent with the model. We examined *jaw-D*-like models in which the non-uniform growth across the mediolateral axis was symmetric or asymmetric, both of which generated curvature (Fig 8I and 8J). These simulations captured the variations in the *jaw-D* leaves, which range in their amount of asymmetry under our constrained growth conditions (S4–S6 Fig). Thus, in wild type, within the basipetal growth gradient, growth is carefully coordinated across the mediolateral axis to avoid growth conflict such that the leaf remains flat. Conversely, lack of coordination across the mediolateral axis in *jaw-D* leads to curvature.

We also examined growth rates along the mediolateral axis to determine whether growth is similarly coordinated in the perpendicular direction. In both wild type and *jaw-D*, the midrib grows slower in the mediolateral direction than the blade (S15 Fig). However, in both wild type and *jaw-D*, the mediolateral growth is coordinated all the way along the proximal–distal axis at each point. Thus, mediolateral growth coordination is maintained in the proximal–distal direction, suggesting this coordination contributes to the flatness of wild-type leaves.

### Simulations of growth patterns show that curvature develops when growth is uneven across the leaf in the direction perpendicular to the main direction of growth

**Fig 9:**
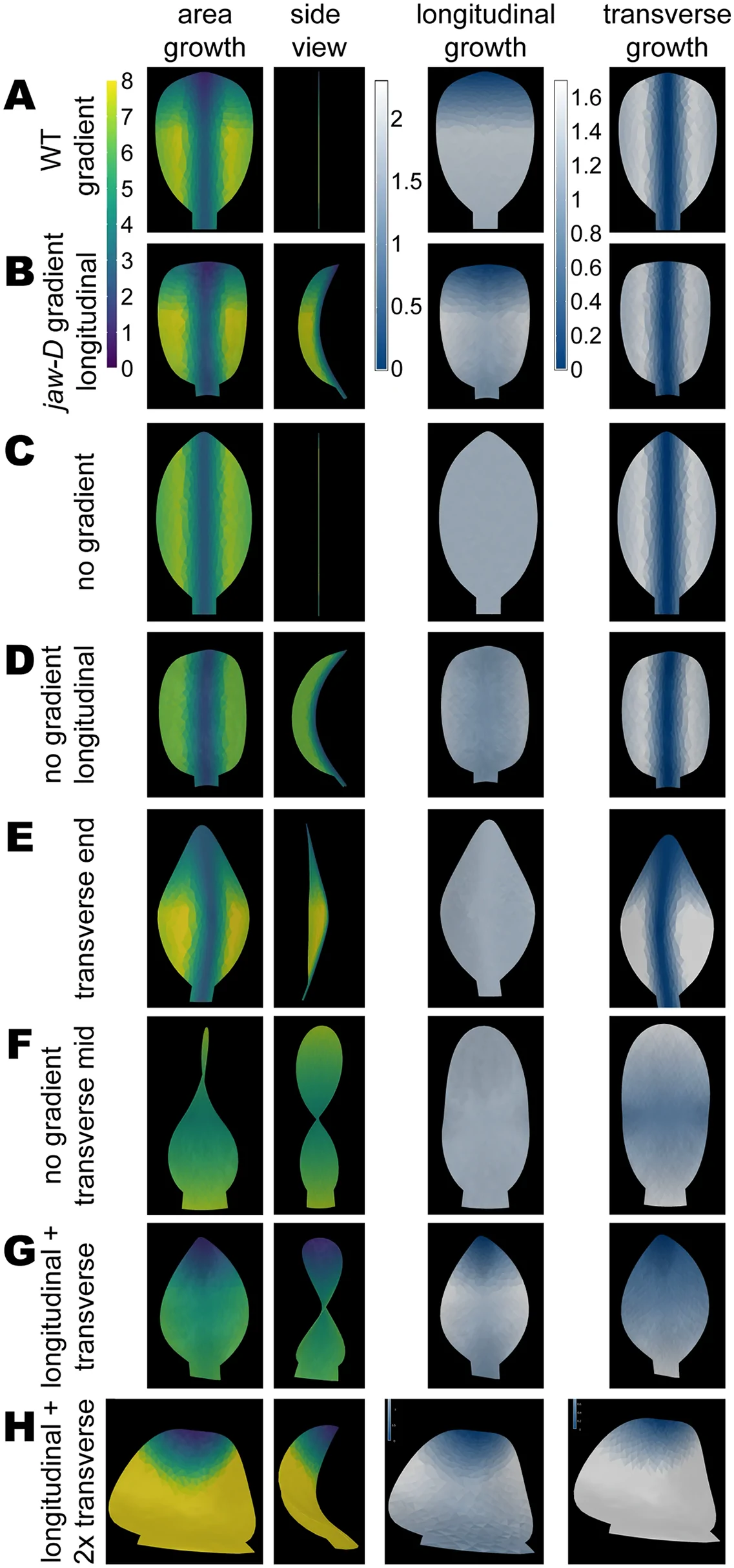
Modeling studies of growth patterns show that curvature develops when growth is uneven across the leaf in the direction perpendicular to the main direction of growth. Each row shows one model with different heatmaps quantifying the growth patterns. From left to right, the first column shows the areal growth rate. The second column shows the leaf from the side to display the curvature. The third column shows the growth rate along the longitudinal axis. And the fourth column shows the growth rate along the transverse axis. All growth rates are ratios of the later time point divided by the earlier time point minus one (such that no growth is 0). The longitudinal direction is the proximal–distal axis of the leaf and the transverse direction is the mediolateral axis of the leaf. **(A)** Model of the wild-type growth pattern with the basipetal growth gradient in the longitudinal direction and slow midrib expansion in the transverse direction. This is the same model shown in Fig 8E and 8F, reproduced here for comparison. **(B)** Model of the *jaw-D* growth pattern with the basipetal growth gradient, the slow midrib, but also the longitudinal growth is uncoordinated in the mediolateral direction with increased growth on the edges and lower growth in the middle, causing curvature. This is the same model shown in Fig 8G and 8H, reproduced here for comparison. **(C)** Model of the wild-type growth pattern, removing the basipetal growth gradient, which produces a flat leaf. **(D)** Model of the *jaw-D* growth pattern with uncoordinated longitudinal growth, removing the basipetal growth gradient, which produces similar curvature to *jaw-D*. **(E)** Model with mediolateral growth that is uncoordinated in the longitudinal direction (lower at the tip and higher at the base), causing the leaf to dome. **(F)** Model with mediolateral growth that is uncoordinated in the longitudinal direction (lower in the middle and higher at top and bottom), causing the leaf to twist. **(G)** Model with longitudinal growth that is uncoordinated in the mediolateral axis and transverse growth that is uncoordinated in the proximal–distal axis, which causes twisting of the leaf. **(H)** Model with longitudinal growth that is uncoordinated in the mediolateral axis and 2-fold greater transverse growth that is uncoordinated in the proximal–distal axis, which causes the leaf to form a saddle shape. See S6–S13 Videos. The code for the model is available at https://doi.org/10.17605/OSF.IO/YTVW9.

We next used the model to explore the effect of different growth patterns on the curvature of the leaf. Our modeling results confirm that curvature develops when growth is uneven across the leaf in the direction perpendicular to the principal direction of growth. Thus, to maintain flatness, all regions on any line parallel to the leaf’s mediolateral axis must share the same proximal–distal growth rate. Likewise, all regions on any line parallel to the proximal–distal axis must share the same mediolateral growth rate. Thus, our model of the wild-type growth pattern with the basipetal growth gradient in the longitudinal direction and a midrib that expands slowly in the transverse direction produced a perfectly flat leaf (Fig 9A; S6 Video). The basipetal growth gradient does not produce any curvature because all regions across the model leaf’s mediolateral axis grow equally. Likewise, the slow growth of the midrib in the transverse direction does not cause curvature because all regions across the leaf’s proximal–distal axis grow equally. In contrast, the *jaw-D* model generates curvature because the growth in the proximal–distal axis is higher on the edges of the leaf than in the middle (Fig 9B; S7 Video). This unequal growth across the mediolateral axis generates a growth conflict and curvature. The *jaw-D* model also includes the basipetal growth gradient and the midrib, similar to wild type.

We tested whether the basipetal growth gradient has any effect on curvature, by removing it from the wild-type and *jaw-D* models. The removal of the basipetal growth gradient had no effect: the wild-type model remained flat (Fig 9C and S8 Video) and the *jaw-D* model remained curved (Fig 9D and S9 Video). This supports our conclusion that the basipetal growth gradient does not affect curvature because the growth perpendicular to it along the mediolateral axis is uniform across the leaf at each location. Furthermore, our results suggest that changes in the basipetal growth gradient in the *jaw-D* mutant cannot explain the curvature phenotype.

We then asked whether mediolateral growth that was uncoordinated along the proximal–distal axis would also cause curvature. We generated two models of mediolateral growth that were uncoordinated across the proximal–distal axis. The first model with slow mediolateral growth at the tip that increased toward the base of the leaf produced a domed leaf shape (Fig 9E and S10 Video). The second model with fast mediolateral growth in the base and tip of the leaf and slow mediolateral growth in the middle twisted (Fig 9F and S11 Video). This model rotated the *jaw-D* coordination defect by 90°. Thus, lack of mediolateral growth coordination across the proximal–distal axis is sufficient to cause growth conflicts and curvature.

Finally, we asked how uneven growth in both the proximal–distal and mediolateral axis together influenced curvature. When growth was equally uncoordinated in both axes, the model generated a twisted form (Fig 9G and S12 Video). When growth was twice as uncoordinated in the mediolateral axis as in the proximal–distal axis, the model leaf produced a curled saddle shape similar to a potato chip (Fig 9H and S13 Video). Thus, incompatible growth causes twisting, while strong differences in incompatible growth rates can generate a saddle shape. Altogether our models suggest that a lack of growth coordination on any axis perpendicular to the primary direction of growth generates growth conflicts and curvature.

### Differential growth between the adaxial and abaxial sides of the leaf is similar in wild type and *jaw-D*

Leaf curvature is typically attributed to differential growth between the adaxial and abaxial tissues [20,21,61]. We have primarily focused on the abaxial side, since imaging the adaxial side of the leaf is challenging because it is completely obscured by the second leaf and the presence of trichomes, which reduce visibility of the epidermal cells. Nevertheless, we have developed a new protocol to image the adaxial side of the leaf for one day of growth to determine whether an imbalance in adaxial and abaxial growth contributed to the *jaw-D* leaf waviness. We also wondered whether similar defects in growth coordination were occurring both abaxially and adaxially in *jaw-D*.

**Fig 10:**
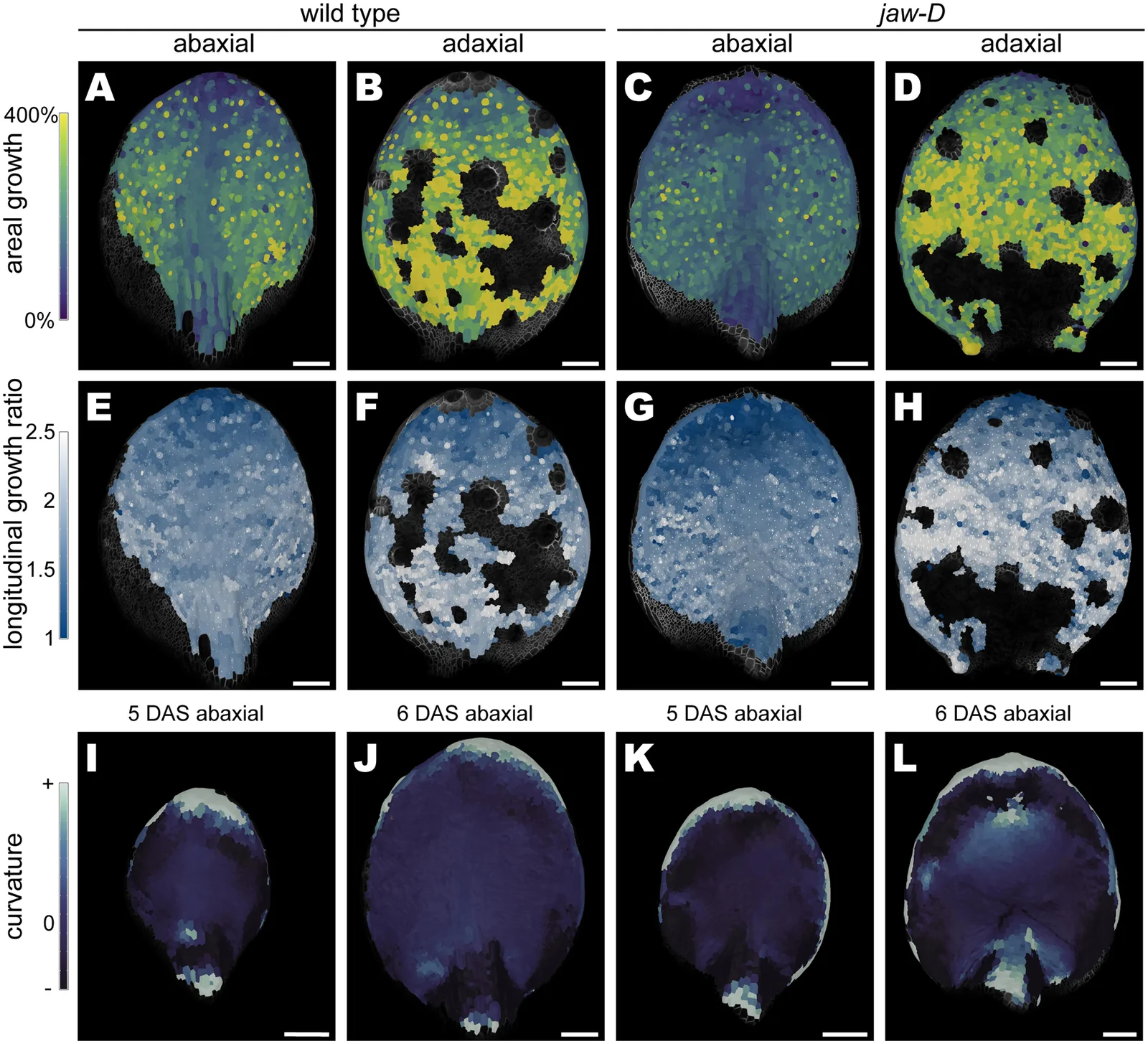
Differential growth between the adaxial and abaxial sides of the leaf is similar in wild type and *jaw-D.* **(A–D)** Areal cell growth heatmap showing the percent growth of the cell over 24 hours from 5 to 6 DAS for the abaxial (A, C) and adaxial (B, D) sides of the same leaf for wild type (A, B) and *jaw-D* mutant **(C, D)**. Note that the growth of the adaxial side is higher than the abaxial side for both wild type and *jaw-D*, consistent with the unfurling of the leaf at this stage. The holes in the segmentation on the adaxial side are due to regions obscured by trichomes. **(E–H)** The cell growth ratio (later time point divided by earlier time point, unitless) along the longitudinal (proximal–distal) axis of the leaf. The principal direction of growth is shown as a small white line in each cell. The abaxial (E, G) and adaxial (F, H) sides of the same leaf for wild type (E, F) and *jaw-D* (G, H) are shown. Note that longitudinal growth is coordinated all the way across the medio-lateral axis of the abaxial side of wild type, but not of *jaw-D*. The growth on the adaxial side is too obscured by trichomes to examine coordination closely. **(I–L)** Gaussian curvature of the abaxial side of the wild type (I, J) and *jaw-D* at 5 DAS (I, K) and 6 DAS **(J, L)**. In this stage, *jaw-D* is just starting to curve. Scale bars = 100 µm. See S16 Fig for additional replicates. The data underlying this figure can be found at https://doi.org/10.17605/OSF.IO/YTVW9.

In both wild-type and *jaw-D* leaves, the adaxial side grows faster than the abaxial side, which is consistent with the unfurling of the leaf at this stage (Figs 10 and S16). However, there are no obvious differences in rates of adaxial growth between the wild type and *jaw-D*, suggesting this is not the cause of the abnormal curvature in the *jaw-D* leaf. The defect in coordination of proximal–distal growth rates across the mediolateral axis on the abaxial side is still evident in *jaw-D* leaves and there are hints of a similar defect in growth coordination on the mediolateral axis of the adaxial side (Figs 10 and S16). However, the large gaps in analyzed cells due to the trichomes obscuring the cells prevent us from making a strong conclusion in this regard. Overall, our results support the conclusion that curvature in *jaw-D* mutants is caused by a lack of coordination in proximal–distal growth across the mediolateral axis, rather than by differential growth between the adaxial and abaxial tissues.

## Discussion

**Fig 11:**
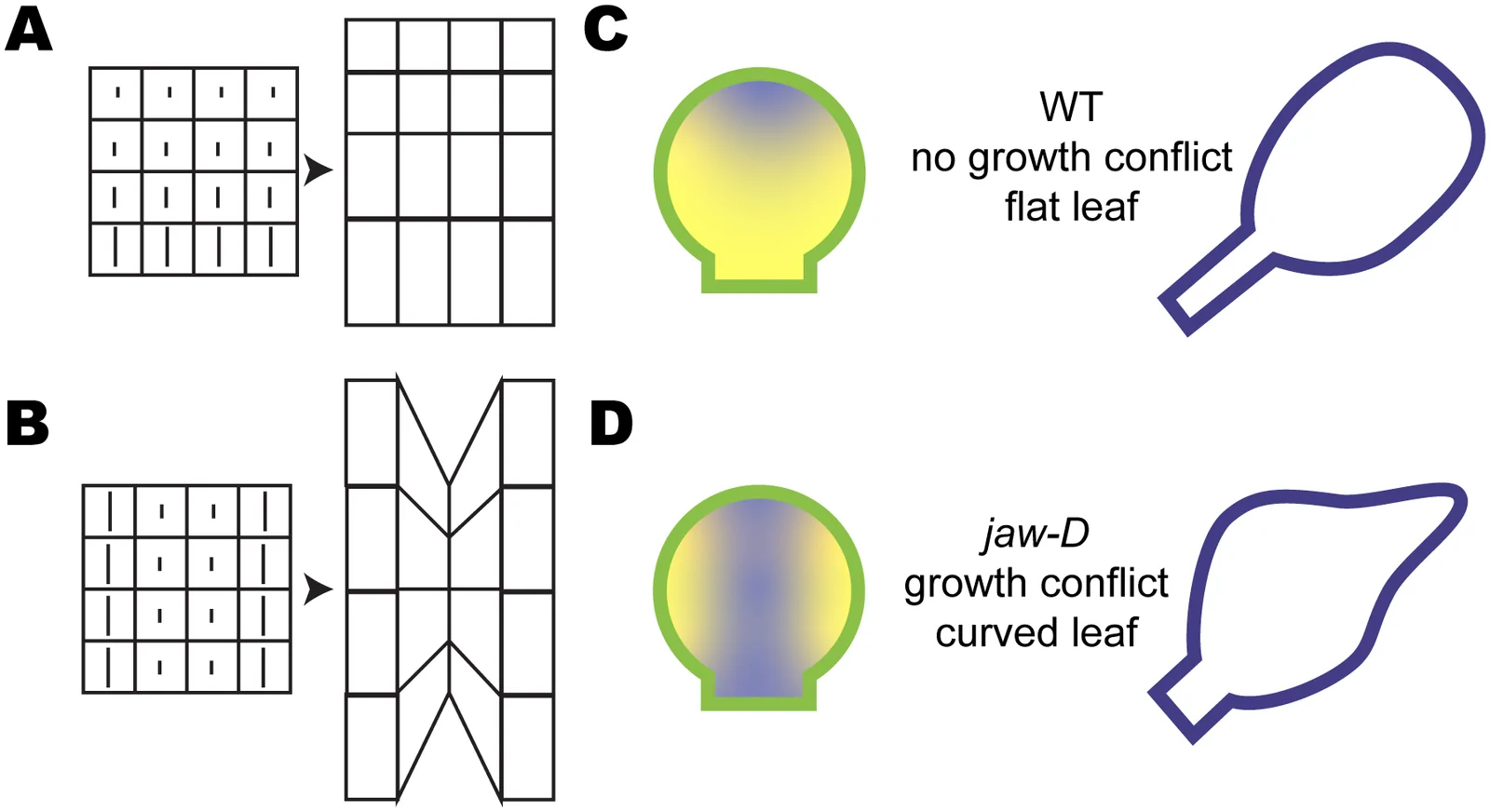
Cell growth is coordinated across the medial–lateral axis of the leaf to maintain leaf flatness in WT while medial lateral growth conflicts lead to curvature in *jaw-D.* **(A)** Conceptual diagram illustrating that as long as growth rates are coordinated all the way across the width of the tissue, no growth conflicts are created, despite a basipetal growth gradient with slower-growing cells at the top. **(B)** Conceptual diagram illustrating that different cell growth rates across the width of the leaf create growth conflicts that will lead to curvature. **(C)** The basipetal growth gradient where slower growth (purple) starts at the tip of the leaf and descends maintains coordination of growth rates across the width of the leaf and thus produces flat leaves. **(D)** The slower growth of the midrib (purple) than the leaf blade (yellow) in *jaw-D* creates growth conflicts causing the leaf to curve. In addition, the basipetal growth gradient is flattened in *jaw-D*.

With this work, we show that coordination of growth rates across the width of wild-type leaves prevents the formation of growth conflicts, allowing the leaves to develop flat blades (Fig 11A and 11C). Live imaging and modeling reveal that disruption of this growth coordination across the width of the *jaw-D* leaf, particularly the slower growth of the midrib relative to the blade, causes *jaw-D* leaves to curve (Fig 11B and 11D). By conducting live cellular imaging on the same leaves from first initiation through a 400-fold increase in area and 100-fold increase in cell number over six days, we have captured an immense level of detail of this process (Fig 1). We found that disruptions in tissue curvature in *jaw-D* become strong at 6 DAS (Fig 2). The *jaw-D* leaves exhibit two major disruptions to the typical wild-type pattern of cellular growth. First, whereas wild type exhibits a basipetal growth gradient with a proximally localized growth maximum and slowing growth gradient towards the tip, the *jaw-D* leaf exhibits a pattern of medium growth broadly extending along most of the length of the leaf, thus spatially flattening the basipetal gradient (Figs 3 and 4). While growth is greater on the adaxial side of the *jaw-D* leaf than the abaxial side, this is also true of wild type and is consistent with the unfurling of the leaf, thus, it is unlikely to be contributing to the overall waviness of *jaw-D* (Fig 10). Corresponding with the progressive basipetal slowing of growth, there is a basipetal wave of cell differentiation as shown by the lobing of pavement cells in wild type, which is delayed and reduced in *jaw-D* (Figs 6 and 7). Second, the wild-type growth rate is relatively uniform across the width of the leaf at each height, whereas the *jaw-D* leaf exhibits slower growth at the midrib than the leaf blade (Figs 3–4, and 8). Thus, the wild type avoids growth conflicts, whereas they are created in *jaw-D*. Finally, we used modeling to test whether the slower growth of the midrib from the blade in *jaw-D* leaves generates the curvature of the leaf (Figs 8 and 9). Simulations of this growth change reveal that uniform proximal–distal cell growth rates across the width of the leaf are essential for maintaining flatness in wild type and unequal proximal–distal cell growth rates between midrib and blade generate positive curvature in *jaw-D* (Figs 8 and 9). Overall, we conclude that cell growth is coordinated across the width of the wild-type leaf to maintain flatness of the leaf blade.

The alterations in growth of the *jaw-D* mutant are more complex and nuanced than expected based on current understanding of the molecular function of *miR319*. The *jaw-D* mutant phenotype is caused by the insertion of strong 35S enhancer elements upstream of miR319, causing stronger expression of miR319 [33]. In wild type, miR319 is expressed from the base of leaves and floral organs [46] where it targets the mRNAs encoding class II TCP transcription factors, limiting their accumulation to the distal tip of the leaf [33,34]. Class II TCP transcription factors promote cell differentiation and inhibit cell division, contributing to the formation of the basipetal gradient of growth and maturation in wild type. Our growth data agrees well with this model in wild type as a region of fast growth and division is present at the base of wild-type leaves (Figs 3 and 4). Since *jaw-D* over-expresses miR319 and should therefore further decrease the presence of TCPs in the leaf, we expected there to be an increase in the size of the fast growth and division zone seen in wild type. However, the change was more nuanced. Instead, the growth rates were more moderate in *jaw-D* and distributed further along the proximal–distal axis. *jaw-D* also affected the midrib versus blade growth rates, leading to a lack of coordination. We noted that cell divisions followed the altered growth pattern in *jaw-D,* and that their contribution to the organ ends earlier than growth. We also noticed an interesting difference between cellular differentiation programs. Namely, the basipetal differentiation of pavement cells is altered in *jaw-D*, while the initiation and spacing of stomata remain intact (Fig 7). Our results highlight the diversity of differentiation programs present in leaves and how high-resolution spatial and temporal imaging can help to resolve the different contributions of these independent processes to leaf form and function.

In many organisms, mechanical instabilities can induce organ waving or folded forms during normal development [62]. Studies in kelp suggest that the water flow stresses experienced by the same species in different environments determine blade ruffling or flattening [22]. In animals, intestinal and cranial folding out of the plane have been attributed to differential growth and buckling between layers [63–67]. Work in *Drosophila* also concurs that the spatial distribution of differential growth is important in creating multiple folds of the wing disc epithelium [24]. Our model complements recent observations that spatially specific reinforcement of tissue stresses occurs in three dimensions to promote the initial flattening of the rounded leaf primordium to form a lamina [68]. Likewise, cell wall integrity sensing and proper response to mechanical signals have recently been found to be required for maintaining the flatness of the thallus in the liverwort *Marchantia polymorpha* [69].

Additionally, while many leaves from different species exhibit a similar basipetal growth gradient as Arabidopsis, leaves from some species exhibit acropetal (growth slowing progressively from base to tip), diffuse (growth throughout) or bidirectional (growth from the center of the leaf) growth patterns. The microRNA miR396, which represses growth by inhibiting *growth-regulating factors* (GRFs), has been shown to be present in basipetal, acropetal and uniform patterns along the proximal–distal axes of different plant species [13]. It would be interesting if the growth patterns we measured had corollaries in these species where the miR396 pattern differs from Arabidopsis. We predict these cases would follow our general mechanism—that the proximal–distal growth across the width of the leaf at each height remains uniform to ensure development of a flat blade, though the specific distributions of growth rate zones may vary. As our study highlights, future work on leaf flatness should focus on the long-distance molecular and mechanical mechanisms coordinating cellular growth across the width of the leaf to prevent growth conflicts and maintain flatness.

## Materials and methods

### Plant growth conditions

For live imaging, seeds were surface sterilized by washing once with 70% EtOH, 0.05% SDS solution for 7–10 min on a nutating shaker, then 3 subsequent washes with 100% EtOH, then dried on autoclaved filter paper. Seeds were sown on 0.5x Murashige and Skoog media (pH 5.7, 0.5 g/L MES, 1% phytoagar) supplemented with 1% sucrose. For genetics, plants were grown to maturity on Lambert Mix LM-111 soil. All plants were stratified for at least 2 days at 4 °C then transferred to Percival growth chambers and grown under 24-hour fluorescent light, ∼100 μmol m^−2^ s^−1^ at 22 °C.

### Plant accessions

Wild-type plants are ecotype Col-0. *jaw-D* leaves are the *jaw-1D* allele from [33]. Plants were crossed with the epidermal-specific fluorescent reporters for plasma membrane (pAR169 *AtML1:mCitrine-RCI2a*) and nucleus (pAR229 *AtML1:H2B-TFP*) [70,71]. In subsequent generations, plants homozygous for both markers were selected. These lines have been deposited in the ABRC under accession numbers CS73343 (wild type pAR169 pAR229) and CS73344 (*jaw-D* pAR169 pAR229). Note that only the plasma membrane marker was imaged for the purposes of this paper.

### Sample preparation—Live imaging

Seedling germination times have inherent variability [72]. In our hands, we found that the first two leaves of wild-type and *jaw-D* plants initiated three days after being exposed to our growth conditions (reported as Days After Sowing—DAS). At this stage, the cotyledons are fully greened and have bent at 90° angles from the stem. Therefore, we dissected cotyledons off the early seedlings 2 DAS, allowed the plants to rest for one day and then proceeded to capture images of growing first leaves using lines expressing the plasma membrane marker. Coverslips were suspended a few millimeters above the sample with vacuum grease to encourage leaves to remain in good imaging positions. Samples beneath coverslips were submerged in perfluorodecalin, a high refractive index compound that allows for proper gas exchange during imaging. More detail about our imaging method can be found in our publication [52].

### Confocal imaging

Plants were imaged on a Zeiss 710 upright laser scanning confocal microscope with 20× Plan-Apochromat NA 1.0 water immersion lens, with z-step between 0.5 and 1.0 µm. The plasma membrane mCitrine marker was excited with a 514 nm wavelength laser at 1%–2% power and the emission spectra from 519–650 nm was collected. As samples grew and were too large to image in one stack, separate scans were taken and stacks manually aligned and merged in MorphoGraphX 2.0 [53].

### Fully grown leaf imaging

First and second leaves were harvested from plants once fully grown, at least 31 DAS, and scanned with a Canon CanoScan LiDE 110 scanner.

### Image processing—Live imaging

Zeiss.LSM imaging files were converted to TIF in ImageJ. Tiff files were imported into MorphoGraphX 2.0 [53]. The MorphoGraphX image processing pipeline has been described in detail [52]. Briefly, masks were created of the confocal stacks through 1–3 rounds of Gaussian blurring, then edge detection, closing holes in older samples where masks showed gaps, surface creation, manual selection and deletion of the surface that did not contain confocal signal and then projection of the confocal signal onto the surface. Meshes were subdivided once, then subject to 2–3 rounds of auto-segmentation, adaptive mesh subdivision at the new cell borders and projection of the confocal signal back onto the refined mesh. Cell segmentations were manually corrected immediately after segmentation or through the process of manual cell lineage tracing and cell junction correction using the check correspondence process. Cell and growth parameters were measured and heatmaps generated using a python script run through MorphoGraphX 2.0. All >77,000 cells in the final analysis were selected based on the presence of lineage tracking ensuring that only cells that were manually curated were included in the final dataset. All segmented meshes and measures are included at https://doi.org/10.17605/OSF.IO/D7X3Y).

### Image processing—Fully grown leaves

Scanned leaf images were processed using the ROI tool in ImageJ. Each leaf was first outlined with the white paintbrush tool to color over bits of dirt and shadows. Images were then converted to 8-bit grayscale and a threshold of 187 was applied. To measure leaf area, each leaf outline was selected with the wand tool, filled in using the Ctrl+F shortcut and measured with the Ctrl+M shortcut. The whole leaf and lamina length were measured using the segmented line tool by tracing a line from tip to base of the lamina and then along the middle of the petiole. The lamina was then digitally separated from the petiole by drawing over the petiole neck with the paintbrush tool. The separated lamina was selected with the wand tool and area measured with Ctrl+M. Petiole length and area were determined by subtracting the measured lamina values from the whole leaf values. Ripples were counted as protrusions visible in the thresholded images that were symmetrical along the proximal–distal axis (see Fig 1K). Bulges on one side were counted as half ripples.

### Data analysis

All data processing, analysis and plotting was performed in RStudio [73,74]. Sigmoidal models were fit using [75]. CVs were compared using [76]. Scripts used to process the data and create each figure can be found at https://github.com/kateharline/jawd-paper. We have provided the segmented meshes, measures of cellular growth, divisions and characteristics to enable further exploration into the variety of cell lineages we captured in this data set available at osf.io (https://doi.org/10.17605/OSF.IO/D7X3Y).

### *Adaxial*–*abaxial live imaging and growth analysis*

After germination, the cotyledons were removed as previously described to keep this experiment as much in line with the abaxial-only live imaging as possible as well as to reduce the amount of stress the seedling would experience at once. Please note that these seeds germinated three days after stratification instead of the two days after stratification in the abaxial-only live imaging experiment. Therefore, we have subtracted 1 DAS to calibrate the timing and ensure clear communication of which developmental stage the leaves are comparable.

After removing the cotyledons, the plants were allowed to grow for an additional day, after which one of the first two true leaves was dissected off and the seedling was left to recover for one day before imaging began. Which leaf was dissected off was primarily based on which one could be more easily removed. The dissection followed a similar procedure as dissecting the cotyledons. Tweezers were used to gently hold the hypocotyl to keep the seedling in place. Then a needle was used to carefully remove one of the leaves without damaging the other. After this dissection, the remaining leaf was checked under the dissecting scope to determine if it was damaged. The following day during imaging, maximum intensity projections of the leaves were checked to determine if there was damage that was missed the previous day. Leaves were quality checked like this throughout imaging.

Overall, imaging followed a similar procedure as described previously. Once all the leaves on a plate had been imaged on the first side, the plate was then taken to the dissecting microscope, where the leaves were turned over before imaging the other side of each leaf. For the abaxial side, a step size of 1 µm was used, for the adaxial side, a step size of 0.5 µm was used. This was done due to the trichomes on the adaxial side blocking signal. A finer step size increased the resolution of the image, allowing visualization of more of the cells.

The stacks were merged in MorphoGraphX, and the trichomes on the adaxial side were manually deleted by using the voxel edit tool. Much of the mesh creation for the adaxial and abaxial sides was similar to the previous section; however, for the adaxial side, when creating the mesh, the processes “Stack/Filters/Top Hat”, “Stack/Filters/Trim High Low Values” and “Stack/Filters/Sharpen Stack” were used in addition to those previously described. Instead of using the Gaussian blur, the process “Stack/Filters/Median” was used to reduce the noise of the stack in preparation for creating the mesh. For the process “Stack/Morphology/Edge Detect” the threshold was reduced to 3,000. Additionally, for areas where there was no signal (where the trichomes were located), these spaces were manually filled in by using the voxel tool. They were filled in just enough that the gaps would be closed by the “Stack/Morphology/Closing” process. After these were added in the areas with no signal, they were trimmed to be the same size as the surrounding area. This was done to create a smooth mesh for analysis. After the mesh was created and the signal from the stack was projected onto it, these areas were double-checked to ensure there was no signal in the area. After these areas were filled in, the process “Stack/Morphology/Closing” was run with the values of 10 for the X radius, 20 for the Y radius, and 6 for the Z radius (double of what was run for the abaxial side). These values were higher to account for the greater number and area of holes that needed to be closed on the adaxial side versus the abaxial side, courtesy of the trichomes.

The rest of the mesh-making, segmentation and analysis followed the same method for abaxial and adaxial sides of the leaf. The fully segmented and parent-tracked meshes are available at osf.io (https://doi.org/10.17605/OSF.IO/YTVW9).

### One-dimensional model of leaf length

We developed a one-dimensional discrete-time model to capture the leaf length dynamics for WT and *jaw-D* genotypes. Our goal was to explore how contrasting spatial growth profiles for WT and *jaw-D* genotypes could lead to overall similar leaf length dynamics. The model considered a spatio-temporal cellular growth rate at any position along the leaf length and over time, and used it in a discrete-time model setting to compute the length across days. For technical details, please refer to S1Text.

### Analysis of local temporal and spatial variability in cell growth

To quantify local temporal and spatial variability in cell growth, we followed [55]. The areal growth rate of a cell between two consecutive time points was defined by ${AGR}_{\text{1}}={(A}_{\text{2}}/A_{\text{1}})/t\;$, where $A_{\text{1}}$ and $A_{\text{2}}$ denoted the cell area in the first and second time point, respectively, and t was the elapsed time between the time points measured in hours. If the cell divided between the two time points, $A_{\text{2}}$ was the sum of the areas of all daughter cells. Local temporal variability in cell area growth was measured using the formula $D_{area}=\frac{\left|AGR_{\text{1}\;}-AGR_{\text{2}}\right|}{\left|AGR_{\text{1}}+AGR_{\text{2}}\right|}\;$. Since this measure was calculated based on 3 consecutive time points, it was ambiguous for which time point we were measuring temporal variability. To resolve this ambiguity, we always used the formula to measure the temporal variability at the second (middle) time point. Given a cell c the local spatial variability of the cell was measured by using its areal growth rate AGR(c) and the areal growth rates of its bordering neighbors AGR(i), i = 1,2, ..., k, where k denotes the number of bordering neighbors. Local spatial variability in cell area growth was measured using the formula $V_{area}=\sum_{i=\text{1}}^{k}\frac{\left|AGR(c)\;-AGR(i)\;\right|}{\left|AGR(c)\;+AGR(i)\;\right|}\;$. The local spatial variability was only calculated based on two time points and the formula was used to measure the variability at the first time point.

### Cellular Fourier analysis

Below we summarize the main steps of the spectral analysis. For more technical details, refer to S1 Text and [57].

We analyzed the areal growth rate and the deformation of tissues. The areal growth rate of each cell was defined as the relative difference of their surface areas at successive time steps divided by the time delay between two time steps. The deformation was a symmetric matrix summarizing the two principal directions of growth, and the ratios to apply in these two directions to deform a cell’s contour at a preceding time step to the same cell’s contour at a following time step.

We then performed the spectral analysis consisting of representing the spatial variation of the areal growth or of the deformation matrix as a linear combination of harmonics. Each harmonic was associated with a frequency characterizing its spatial variation scale. By performing our analysis, we obtained the Cellular Fourier Transforms of the areal growth rate and of the deformation. These were the amplitudes with which the different harmonics decompose the areal growth rate and the deformation matrix. For a tissue, the first harmonic was a constant associated to the spatial frequency 0, and the corresponding CFT for the areal growth rate was the whole tissue’s areal growth. The two following harmonics varied at the scale of the tissue and the associated CFTs for the areal growth rate gave the average growth gradient at the tissue scale. The higher the rank of the harmonics was, the higher the spatial frequency and the smaller the considered space scale were. The deformation being described by a matrix, its CFTs were also matrices. The first CFT will, for example, be the average tissue deformation. We noticed that, although a cell’s deformation was 2D and lay in its tangential plane, the overall tissue deformed in 3D if it was not flat. The CFTs of the deformation matrix depended therefore on the tissue geometry. To analyze growth anisotropy, we considered the difference between the first two eigenvalues of the CFTs of deformation which we divided by the time delay between two time steps. It quantified the deviation from isotropic deformation.

The spectra we finally considered were those of the areal growth rate and the deviation from isotropic growth. These spectra were smoothed to be comparable among different tissues. In these spectra, the y-axis represented the CFTs and the x-axis represented spatial frequencies. Low frequencies corresponded to large spatial variation scales and high frequencies to small spatial variation scales. Spatial frequencies equal to one corresponded to the cell scale (mean cell size).

### Finite element method simulations

The template for the growing leaf model was created from an initial flat 2D contour representing the young leaf shape. This was triangulated and then extruded into a sheet made of a single layer of wedges (prisms). This follows the structure of previous models of leaf shape [8], although in our model a small amount of noise was added to the vertices in the normal direction allowing the structure to bend out of the plane. The MorphoMechanX (https://www.MorphoMechanX.org) modeling framework was used for the FEM simulation [77–79]. An isotropic linear material model was used. Growth was implemented by increasing the size of the reference configuration of the elements, with independent control of growth parallel or perpendicular to a growth axis. The rates of growth and the growth direction were specified manually through the software interactive GUI based on qualitative assessment of live imaging data. After each growth step, the mechanical equilibrium was found, and residual stresses were released.

The mathematical details about mechanical equilibrium calculation and growth algorithm are provided together with the simulation models in S1 Text.

## Supporting information

S1 Fig WT leaf live imaging replicate 1. Higher magnification image of main text 8 DAS WT leaf (left) and its corresponding, to scale, 3 DAS primordia (right). Black bar = 200 µm. Associated with Fig 1. The data underlying this figure can be found at https://doi.org/10.17605/OSF.IO/D7X3Y.
(TIF)

S2 Fig WT leaf live imaging replicate 2. Higher magnification image of replicate 8 DAS WT leaf (left) and its corresponding, to scale, 3 DAS primordia (right). Black bar = 200 µm. Associated with Fig 1. The data underlying this figure can be found at https://doi.org/10.17605/OSF.IO/D7X3Y.
(TIF)

S3 Fig WT leaf live imaging replicate 3. Higher magnification image of replicate 8 DAS WT leaf (left) and its corresponding, to scale, 3 DAS primordia (right). Black bar = 200 µm. Associated with Fig 1. The data underlying this figure can be found at https://doi.org/10.17605/OSF.IO/D7X3Y.
(TIF)

S4 Fig *jaw-D* leaf live imaging replicate 1. Higher magnification image of main text 8 DAS *jaw-D* leaf (left) and its corresponding, to scale, 3 DAS primordia (right). Black bar = 200 µm. Associated with Fig 1. The data underlying this figure can be found at https://doi.org/10.17605/OSF.IO/D7X3Y.
(TIF)

S5 Fig *jaw-D* leaf live imaging replicate 2. Higher magnification image of replicate 8 DAS *jaw-D* leaf (left) with less visual skew and its corresponding, to scale, 3 DAS primordia (right). Black bar = 200 µm. Associated with Fig 1. The data underlying this figure can be found at https://doi.org/10.17605/OSF.IO/D7X3Y.
(TIF)

S6 Fig *jaw-D* leaf live imaging replicate 3. Higher magnification image of replicate 8 DAS *jaw-D* leaf (left) and its corresponding, to scale, 3 DAS primordia (right). Note the petiole broke due to the strong curvature. Black bar = 200 µm. Associated with Fig 1. The data underlying this figure can be found at https://doi.org/10.17605/OSF.IO/D7X3Y.
(TIF)

S7 Fig Additional features of live-imaged and fully grown leaves. **(A)** Leaf width increases exponentially in WT (squares) and *jaw-D* (circles) leaves during the live imaging experiment. Colors indicate DAS. **(B)** Final leaf area of fully grown first and second leaves between WT and *jaw-D* is not significantly different, though the distributions are distinct with WT showing a bimodal distribution and *jaw-D* with a more common median value and more extreme values. Color, shape and line type indicate genotype (solid, squares, dark gray = WT; dotted, circles, light gray = *jaw-D*). **(C)** Total leaf length of fully grown first and second leaves between WT and *jaw-D* shows a similar pattern with slightly shorter leaves in *jaw-D,* likely due to petiole length differences only (p < 0.05, Wilcox test). **(D)** Median length of the lamina measured from the petiole insertion point to the distal tip of the leaf is not significantly different between fully grown WT and *jaw-D* leaves (*p* > 0.05, Wilcox test). **(E)** The median relative length between the first and second leaves within the same plant of WT and *jaw-D* is not significantly different, though *jaw-D* exhibits much more extreme values (*p* > 0.05 Wilcox test). **(F, G)** The logarithm of leaf length (F) and leaf area (G) with plotting as in (A) demonstrate that these values are increasing exponentially over the live imaging experiment. Associated with Fig 1. The data underlying this figure can be found at https://doi.org/10.17605/OSF.IO/D7X3Y, and the R code to create the graphs from the data is available at https://doi.org/10.17605/OSF.IO/YTVW9.
(TIF)

S8 Fig *jaw-D* leaves have high Gaussian curvature while wild-type leaves flatten toward 0 Gaussian curvature. **(A, B)** Quantification of the distribution of curvature along the proximal–distal (A) and mediolateral (B) axes for all three replicates of WT (top, squares, solid lines) and *jaw-D* laminas (bottom, circles, dashed lines). A proximal–distal gradient is evident starting at 6 DAS in *jaw-D* leaves (orange arrow), whereas WT flattens starting at 7 DAS (orange arrows). In the mediolateral direction, wild type flattens starting at 7 DAS (orange arrows), whereas *jaw-D* has broad lateral complexity of curvature. Note that, as all leaves flatten, the Gaussian curvature changes dynamically over multiple orders of magnitude during the experiment, so the y-axis changes over time. Further, the petiole and margin are sources of extreme outliers in curvature. Therefore, the scale for the heatmaps and graphs representing each time point has been calculated to encompass the 85% range of curvature values around the mean for that time point. Associated with Fig 2. The data underlying this figure can be found at https://doi.org/10.17605/OSF.IO/D7X3Y, and the R code to create the graphs from the data is available at https://doi.org/10.17605/OSF.IO/YTVW9.
(TIF)

S9 Fig Growth and division patterns are consistent between replicates. Distribution of cell growth and division of each cell for 1-day intervals per whole leaf sample of WT (first three rows) and *jaw-D* (last three rows). *n* indicates the number of cells in each panel. The x and y axes represent mediolateral and proximal–distal positions, respectively, normalized between 0 and 1 for the given leaf and time point. Colors indicate 24-hour time points. The size and color intensity of each point represent the number of divisions and areal growth, respectively, a cell has experienced between imaging days. We do not separate cells between the petiole and the lamina, so the proximal–distal normalization is from the base of the petiole. Associated with Fig 3. The data underlying this figure can be found at https://doi.org/10.17605/OSF.IO/D7X3Y, and the R code to create the graphs from the data is available at https://doi.org/10.17605/OSF.IO/YTVW9.
(TIF)

S10 Fig Cell division patterns in WT and *jaw-D.* **(A, B)** Cell divisions over 24-hour intervals versus position along the proximal–distal axis for each cell in all three WT (A, solid lines, squares) lamina replicates or *jaw-D* (B, dotted lines, circles) replicates. Cell divisions are concentrated more distally in *jaw-D* than WT. **(C, D)** Cell divisions over 24-hour intervals versus position along the mediolateral axis for each cell in all three WT (C, solid lines, squares) lamina replicates or *jaw-D* (D, dotted lines, circles). Cell divisions are distributed more similarly between WT and *jaw-D* along the mediolateral axis. Scale bars = 200 µm. Use of density plots, coloring, axis selection and normalization as in Fig 4. Note that density plots at later time points were not calculated if the majority of cells were not dividing. In this case, only points representing individual cells are plotted. Associated with Fig 4. The data underlying this figure can be found at https://doi.org/10.17605/OSF.IO/D7X3Y, and the R code to create the graphs from the data is available at https://doi.org/10.17605/OSF.IO/YTVW9.
(TIF)

S11 Fig Spatio-temporal analysis with stomata excluded. **(A, B)** Quantification of the mean (A) local growth spatial variability or (B) local growth temporal variability for time windows as in main Fig 5C and 5D, with stomata excluded. Excluding stomata does not change patterns of growth heterogeneity observed between WT and *jaw-D.* WT and *jaw-D* local spatial and temporal growth variability are largely indistinguishable, though *jaw-D* replicates are generally tighter around the mean. Associated with Fig 5. The data underlying this figure can be found at https://doi.org/10.17605/OSF.IO/YTVW9.
(TIF)

S12 Fig Additional clonal analysis. **(A–D)** Clonal analysis of WT (top) and *jaw-D* (bottom) clonal sectors mapped from 3 DAS to 8 DAS on sample leaf meshes in shades of purple along the proximal–distal (A) or mediolateral (C) axis. Clonal analysis is as in Fig 6, except that it starts at 3 DAS instead of 4DAS. (B, D) Quantification for all three whole-leaf replicates. WT (left, dark shading, solid lines) and *jaw-D* (right, light shading, dotted lines). Boxes indicate the 50% interquartile range, middle bar represents median, whiskers represent outliers and squares (WT) or circles (*jaw-D)* represent extreme values. Nearly all 3 DAS proximal–distal bins have similar to enhanced results as 4 DAS with significant differences for 8 DAS locations between WT and *jaw-D,* while mediolateral contributions are more mixed (**** = *p* < .0001, ** = *p* < .01, * = *p* < .05, paired Student *t* test and Bonferroni correc*t*ion). Final leaf contributions from WT seem to emerge mostly mid-leaf and a consistent stronger contribution from *jaw-D* lateral domains. Black scale bars = 200 µm. **(E–H)** Plots of all cells at 8 DAS versus their relative proximal–distal (E, G) or mediolateral (F, H) position in the 3DAS (E, F) or 4DAS (G, H) leaf. Bifurcations in *jaw-D* mediolateral positions could indicate tissue growth conflicts that induce rippling (F, H). Associated with Fig 6. The data underlying this figure can be found at https://doi.org/10.17605/OSF.IO/D7X3Y, and the R code to create the graphs from the data is available at https://doi.org/10.17605/OSF.IO/YTVW9.
(TIF)

S13 Fig Quantification of differentiation as measured by lobeyness. **(A, B)** Lobeyness (the perimeter of a cell divided by its convex hull, a measure of pavement cell identity) versus position along the proximal–distal axis for each cell in all three WT (A, squares, solid lines) or *jaw-D* (B, circles, dotted lines) lamina replicates. Lobeyness is initiated at the distal tip of WT cells at 5 DAS and increases while propagating down to the leaf base. Increases in lobeyness are subtler, later and more disbursed in *jaw-D* samples. **(C, D)** Lobeyness is more evenly distributed across the mediolateral axis in WT (C) and *jaw-D* (D) with greater absolute magnitude in WT. Use of density plots, coloring, axis selection and normalization as in Fig 4. Associated with Fig 7. The data underlying this figure can be found at https://doi.org/10.17605/OSF.IO/D7X3Y and the R code to create the graphs from the data is available at https://doi.org/10.17605/OSF.IO/YTVW9.
(TIF)

S14 Fig Quantification of differentiation of stomata. **(A–D)** Quantification of stomatal spacing (the distance in µm from pavement cells to the nearest stomata, the inverse of stomatal density) versus position along the proximal–distal axis (A, B) or the mediolateral axis (C, D) and WT (A, C) and *jaw-D* (B, D). Note that stomata first initiate at 4DAS. Stomatal spacing is largely the same between WT and *jaw-D* along both axes. Stomatal spacing quickly tapers as stomata are initiated and become evenly distributed through the lamina. **(E–H)** Area (E, F) or circularity (G, H) (perimeter^2^/(4π*area)) versus position along the proximal–distal axis for each stomate in all three WT (E, G, squares, solid lines) or *jaw-D* (F, H, circles, dotted lines) lamina replicates. (E) Stomatal area shows a distal maximum with a graded decrease towards the base for WT samples starting at 5 DAS. (F) Stomata in *jaw-D* have more even sizes. Note that, as the stomatal areas cover many orders of magnitude over time, a log scale is used. (G) Stomatal circularity is further from 1 in WT in a graded pattern signifying anisotropic expansion from tip to base. (H) *jaw-D* stomata are rounder throughout the lamina. These differences demonstrate how stomata may be subject to tissue-level anisotropy and maturation processes only in WT while baseline initiation and differentiation for their physiological role is maintained in WT and *jaw-D*. Use of density plots, coloring, axis selection and normalization as in Fig 4. Associated with Fig 7. The data underlying this figure can be found at https://doi.org/10.17605/OSF.IO/D7X3Y, and the R code to create the graphs from the data is available at https://doi.org/10.17605/OSF.IO/YTVW9.
(TIF)

S15 Fig Mediolateral growth is coordinated along the proximal–distal axis. **(A, B)** Heatmap of cell growth along the mediolateral axis of the wild-type (A) and *jaw-D* (B) leaves from 3–4 DAS. High mediolateral extension is colored white (2) and no extension is colored blue (0). The magnitude of growth along this mediolateral axis is also represented by the length of the white line drawn in each cell. Two regions are magnified to make the cellular growth rates visible across the axis of the leaf. Note that the mediolateral growth rates at each proximal–distal region of the leaf are similar, although the tip of the leaf is growing slower due to the basipetal growth gradient. Scale bars = 100 µm. Associated with Fig 8. The data underlying this figure can be found at https://doi.org/10.17605/OSF.IO/D7X3Y.
(TIF)

S16 Fig Differential growth between the adaxial and abaxial sides of the leaf is similar in wild type and *jaw-D.* From left to right the columns show: (1, 2) Areal cell growth heatmap showing the percent growth of the cell over 24 hours from 5 to 6 DAS for the abaxial (1) and adaxial (2) sides of the same leaf. (3, 4) The cell growth ratio (later time point divided by earlier time point, unitless) along the longitudinal (proximal–distal) axis of the leaf on the abaxial (3) and adaxial (4) sides of the leaf. The principal direction of growth is shown as a small white line in each cell. (5, 6) Gaussian curvature of the abaxial side at 5 DAS (5) and 6 DAS (6, rightmost column). **(A, B)** Wild-type leaf replicates 2 and 3. **(C–E)** *jaw-D* leaf replicates 2–4. Scale bars = 100 µm. Associated with Fig 10, which shows replicate 1 for both wild type and *jaw-D*. The data underlying this figure can be found at https://doi.org/10.17605/OSF.IO/YTVW9.
(TIF)

S1 Video Clonal lineage of 4 DAS and 8 DAS WT meshes in MGX. Mesh representations of 4 DAS (right) and 8 DAS (left) meshes of sample WT leaf in MorphoGraphX software. The video begins by showing the 4 DAS sample with segmented cells indicated with different colored labels. Zooming out shows relative sizes and morphology changes from 4 DAS to 8 DAS. Parent labels are then displayed on 8 DAS mesh to indicate how cells labeled in 4 DAS mesh contributed to 8 DAS sample. Relatively simple, isotropic cells in an amorphous nub have become lobed pavement cells and stomata while the gross leaf morphology has changed to a distinct petiole and flattened, round blade. Black scale bar = 200 µm. The data underlying this video can be found at https://doi.org/10.17605/OSF.IO/D7X3Y.
(MP4)

S2 Video Clonal lineage of 4 DAS and 8 DAS *jaw-D* meshes in MGX. Representations as in S1 Video for *jaw-D* 4 DAS (right) and 8 DAS (left) sample meshes. 8 DAS mesh in *jaw-D* exhibits enhanced proximal curvature, and smaller, less lobed cells. Clonal patches are also smaller. Black scale bar = 200 µm. The data underlying this video can be found at https://doi.org/10.17605/OSF.IO/D7X3Y.
(MP4)

S3 Video 4–8 DAS clonal lineages with animated growth in WT. Clonal lineages traced from 4 DAS to 8 DAS on example WT mesh. Individual lineages indicated as different label colors. Unlabeled cells could not be traced between all time points. Proximal lineages tend to divide and expand many times and often anisotropically, while distal lineages are more isotropic, expand and divide less. Black scale bar = 200 µm. The data underlying this video can be found at https://doi.org/10.17605/OSF.IO/D7X3Y.
(MP4)

S4 Video 4–8 DAS clonal lineages with animated growth in *jaw-D.* Clonal lineages for a *jaw-D* sample with representation as in S3 Video. All lineages are generally more isotropic in expansion and divide less. The data underlying this video can be found at https://doi.org/10.17605/OSF.IO/D7X3Y.
(MP4)

S5 Video Curvature cross-sections of WT and *jaw-D.* 2.5D mesh representations of WT (left, top) and *jaw-D* (right, bottom) 8 DAS samples arranged in MorphoGraphX software. Meshes overlaid to compare curvature at comparable cross-section locations. Clipping plane (white lines) used to scroll through mesh sections to show proximal curvature increase in *jaw-D*. White scale bar = 200 µm. The data underlying this video can be found at https://doi.org/10.17605/OSF.IO/D7X3Y.
(MP4)

S6 Video Simulation of WT leaf (gradient). FEM simulations of leaf mesh growth as in Figs 8E, 8F, and 9A, representing WT. The left heatmap shows areal growth rate, the middle heatmap shows longitudinal (proximal–distal) growth rate, and the right heatmap shows transverse (mediolateral) growth rate. The basipetal growth gradient has been imposed, but growth across the width is uniform, such that no growth conflicts are created, and the leaf remains flat. Likewise, slower mediolateral growth of the midrib has been imposed, but growth across the length is uniform, such that no growth conflicts are created. The modeling code underlying this video can be found at https://doi.org/10.17605/OSF.IO/YTVW9.
(MP4)

S7 Video Simulation of *jaw-D* leaf (gradient, longitudinal). FEM simulations of leaf mesh growth as in Figs 8G, 8H, and 9B, representing *jaw-D*. The left heatmap shows areal growth rate, the middle heatmap shows longitudinal (proximal–distal) growth rate, and the right heatmap shows transverse (mediolateral) growth rate. The basipetal growth gradient and the slow mediolateral growth of the midrib have been imposed. In addition, proximal–distal growth of the midrib is lower than growth in the blade (symmetric on both sides), which causes growth conflicts and curvature of the leaf. The modeling code underlying this video can be found at https://doi.org/10.17605/OSF.IO/YTVW9.
(MP4)

S8 Video Simulation wild-type leaf without the basipetal growth gradient (no gradient). FEM simulations of leaf mesh growth as in Fig 9C, representing wild type without the basipetal growth gradient. The left heatmap shows areal growth rate, the middle heatmap shows longitudinal (proximal–distal) growth rate, and the right heatmap shows transverse (mediolateral) growth rate. Only slower mediolateral growth of the midrib has been imposed, which does not create a growth conflict and the leaf remains flat. The modeling code underlying this video can be found at https://doi.org/10.17605/OSF.IO/YTVW9.
(MP4)

S9 Video Simulation of jaw-D leaf without basipetal growth gradient (no gradient longitudinal). FEM simulations of leaf mesh growth as in Fig 9D, representing jaw-D without the basipetal growth gradient. The left heatmap shows areal growth rate, the middle heatmap shows longitudinal (proximal–distal) growth rate, and the right heatmap shows transverse (mediolateral) growth rate. Slower mediolateral growth of the midrib has been imposed, and proximal–distal growth of the midrib is lower than growth in the blade (symmetric on both sides), which causes growth conflicts and curvature of the leaf. The modeling code underlying this video can be found at https://doi.org/10.17605/OSF.IO/YTVW9.
(MP4)

S10 Video Simulation of leaf with mediolateral growth that is uncorrelated along the proximal–distal axis at the tip (transverse end). FEM simulations of leaf mesh growth as in Fig 9E. The left heatmap shows areal growth rate, the middle heatmap shows longitudinal (proximal–distal) growth rate, and the right heatmap shows transverse (mediolateral) growth rate. Slower mediolateral growth of the midrib and slower mediolateral growth of the tip of the leaf have been imposed. The uncoordinated mediolateral growth over the proximal–distal axis causes growth conflicts and curvature of the leaf. The modeling code underlying this video can be found at https://doi.org/10.17605/OSF.IO/YTVW9.
(MP4)

S11 Video Simulation of leaf with mediolateral growth that is uncorrelated along the proximal–distal axis (transverse middle). FEM simulations of leaf mesh growth as in Fig 9F. The left heatmap shows areal growth rate, the middle heatmap shows longitudinal (proximal–distal) growth rate, and the right heatmap shows transverse (mediolateral) growth rate. Slower mediolateral growth of the middle of the leaf has been imposed. The uncoordinated mediolateral growth over the proximal–distal axis causes growth conflicts and curvature of the leaf. The modeling code underlying this video can be found at https://doi.org/10.17605/OSF.IO/YTVW9.
(MP4)

S12 Video Simulation of leaf with both uncoordinated proximal–distal and mediolateral growth (longitudinal + transverse). FEM simulations of leaf mesh growth as in Fig 9G. The left heatmap shows areal growth rate, the middle heatmap shows longitudinal (proximal–distal) growth rate, and the right heatmap shows transverse (mediolateral) growth rate. Slower longitudinal growth in the midrib than the margins (similar to *jaw-D*) causes uncoordinated growth across the width of the leaf. In addition, slower transverse growth of the middle of the leaf causes uncoordinated mediolateral growth over the proximal–distal axis. Together these growth conflicts generate a curved and twisted leaf. The modeling code underlying this video can be found at https://doi.org/10.17605/OSF.IO/YTVW9.
(MP4)

S13 Video Simulation of leaf with both uncoordinated proximal–distal and mediolateral growth (longitudinal + 2x transverse). FEM simulations of leaf mesh growth as in Fig 9H. The left heatmap shows areal growth rate, the middle heatmap shows longitudinal (proximal–distal) growth rate, and the right heatmap shows transverse (mediolateral) growth rate. Slower longitudinal growth in the midrib than the margins (similar to *jaw-D*) causes uncoordinated growth across the width of the leaf. In addition, 2-fold slower transverse growth of the tip of the leaf causes uncoordinated mediolateral growth over the proximal–distal axis. Together these growth conflicts generate a saddle-shaped leaf. The modeling code underlying this video can be found at https://doi.org/10.17605/OSF.IO/YTVW9.
(MP4)

S1 Text Supplementary computational details and methods for: (1) One-dimensional model of leaf length; (2) Cellular Fourier transform; (3) Finite element model simulations. The underlying code can be found at https://doi.org/10.17605/OSF.IO/YTVW9.
(PDF)

## Abbreviations

DAS: days after sowing
GRFs: growth-regulating factors
SAM: shoot apical meristem
TCP: teosinte branched 1, cycloidea, proliferating cell nuclear antigen factor 1 and 2.
