# Supplementary material for: Growth-rate coordination across the width of a leaf preserves its flatness
Source: PLoS Biol. 2026 Sep 8;24(9):e3003993. doi: 10.1371/journal.pbio.3003993 (PMC13585336; doi:10.1371/journal.pbio.3003993)
Supplement: Supplementary file 2 [file pbio.3003993.s033.pdf]

Response to reviewers:

We thank the reviewers for their positive and thoughtful comments. We detail our responses to each comment below in blue text.

Reviewer #1: This manuscript investigates the cellular basis of leaf flatness by comparing developing wild-type (Arabidopsis thaliana) leaves with the curved and ruffled leaves of the jaw-D mutant. Through time-lapse live imaging (3-8 days after sowing) combined with finite element modeling, the authors track cellular growth, division, and maturation dynamics. The jaw-D mutant overexpresses miR319, which represses TCP transcription factors and delays leaf maturation.

The study identifies two principal disruptions in jaw-D growth patterns:
(i) the canonical basipetal growth-rate gradient—typically characterized by slow growth at the tip and progressively faster growth toward the base—is flattened, resulting in a more uniform, moderate growth along the entire proximal-distal axis; and
(ii) growth across the width of the leaf (medial-lateral axis) becomes non-uniform, with slower expansion in the midrib region relative to the adjacent blade.

The second point, the failure of mediolateral growth coordination, represents the most critical and unexpected discovery of the study and provides a compelling explanation for the curvature phenotypes observed in jaw-D. The modeling results elegantly demonstrate that proximal-distal growth differences alone cannot account for the curvature, thus revealing that leaf flatness requires coordinated growth across the entire width of the tissue to prevent internal mechanical conflicts.

Furthermore, the authors connect this loss of mediolateral coordination to delayed pavement cell maturation, which compromises the mechanical stability of the tissue. The normal development of stomata serves as an effective internal control, underscoring that the flatness instability arises specifically from the pavement cell domain (and thus mechanics!) rather than from general developmental defects.

Taken together, the integration of live imaging and modeling in this study is truly exceptional—offering a mechanistic depth that is rarely achieved in plant morphogenesis research.

General assessment
This work advances our understanding of how organ flatness is maintained. Whereas previous models of leaf curvature focused primarily on growth differences between the adaxial and abaxial surfaces, this study reveals that coordination of cell expansion across the width of the leaf is equally critical. The authors' demonstration that differential mediolateral growth alone is sufficient to reproduce curvature represents a conceptual shift in how we think about planar organ development.

The study also establishes clear parallels with biomechanical systems in other organisms (e.g., Drosophila wing discs, kelp fronds, animal epithelia) where local growth mismatches generate shape complexity. Overall, the manuscript is well written, methodologically rigorous, and conceptually important. I strongly support publication after revision, provided that the points below are addressed.

Thank you for the positive comments!

Major comments

1. Adaxial-abaxial comparison: In the Introduction, the authors correctly note that leaf curvature is typically attributed to differential growth between adaxial and abaxial tissues. However, the study quantifies growth exclusively on the abaxial side. Have the authors performed, or at least considered, comparable long-term imaging of the adaxial surface? Such data would be essential to determine whether the same growth irregularities (flattened basipetal gradient and non-uniform medial-lateral growth) also occur adaxially. Without this information, the mechanistic link between side-specific growth and curvature remains incomplete. While this limitation does not undermine the main aim of the manuscript, it should be discussed more openly.

We thought the question of what happens to growth on the adaxial side of *jaw-D* was important and interesting, so we developed a new live imaging protocol to image both the abaxial and adaxial side of the leaf in wild type and *jaw-D*. To our knowledge, this is the first time both sides of the leaf have been live imaged and growth analyzed together. To do so, we cut one of the two leaf primordia off, imaged one side of the leaf, flipped it over and imaged the other side, before letting it grow. Given the difficulty of this experiment, we managed to image and analyze leaves for 24 hours (i.e. two time points), which was sufficient to calculate cell growth rates. Unfortunately, the trichomes on the adaxial side obscure the cell growth on the adaxial side substantially, so there are major gaps in the regions of the leaf that we were able to image.

Despite the challenges, the results are interesting and we have included them in a new Figure 10 and Supplemental Figure S16, as well as a new section in the results. In both wild type and *jaw-D* leaves, the adaxial side grows faster than the abaxial side, which is consistent with the unfurling of the leaf (bending backward as the leaf opens) at this stage. There are no obvious differences in the rates of adaxial growth between wild type and *jaw-D*, suggesting this is not the cause of the abnormal curvature in the *jaw-D* leaf. The defect in coordination of proximal distal growth rates across the medio-lateral axis on the abaxial side is still evident in the *jaw-D* leaves. There are hints of a defect in medio-lateral growth coordination on the adaxial side; however, the big gaps in the analyzed cells due to the trichomes, prevents us from making a strong conclusion. Overall, our results are consistent with our finding that it is lack of coordination of growth across the medio-lateral axis that causes the curvature in *jaw-D* mutants.

2. Modeling of leaf length (proximal-distal axis): The 1D model used to simulate leaf length differences between WT and jaw-D approximates the jaw-D growth profile as a constant moderate growth rate along the proximal-distal axis, despite the availability of more detailed

measurements. Since the model concludes that "different proximal-distal growth patterns can yield similar leaf lengths," it would be more convincing to incorporate the experimentally determined jaw-D growth data rather than a simplified constant function. Otherwise, the conclusion risks appearing speculative or selectively supported.

Our modeling goal was to only qualitatively demonstrate that different spatiotemporal growth profiles can result in similar leaf length dynamics over a 30-day period. The model profiles are motivated from data where *jaw-D* shows a flatter growth profile compared to WT based on observation from 3 to 8 days after sowing. To check the robustness of our results, we have now expanded the model to test two different *jaw-D* growth profiles and modified the figure to show changes in the growth profile across developmental time points (Figure 4C-E). Our new figure panels show the growth profiles change in time for each of these model scenarios.

Importantly, the authors should highlight that this and other modeling exercises demonstrate that proximal-distal growth defects alone cannot explain the curvature phenotype—thereby motivating their subsequent focus on mediolateral coordination failure as the physical driver of curvature. Making this logical connection more explicit would greatly strengthen the narrative coherence of the manuscript.

Good point. In the section on the length model, we have added the sentence: “This model hints that proximal-distal growth defects alone cannot explain the curvature phenotype.”

We also added two more leaf growth models (Figure 9C and D) and corresponding results text: “We tested whether the basipetal growth gradient has any effect on curvature, by removing it from the wild-type and *jaw-D* models. The removal of the basipetal growth gradient had no effect: the wild-type model remained flat (Fig 9C; Video S8) and the *jaw-D* model remained curved (Fig 9D; Video S9). This supports our conclusion that the basipetal growth gradient does not affect curvature because the growth perpendicular to it along the mediolateral axis is uniform across the leaf at each location. Furthermore, our results suggest that changes in the basipetal growth gradient in the *jaw-D* mutant cannot explain the curvature phenotype.”

Minor comments

1. Lines 145-157: The authors show that stomatal initiation and petiole formation occur normally in jaw-D, indicating that major developmental programs remain intact. However, the following statement—"Thus, in the first 8 days of leaf development, we have captured key changes in cellular growth that cause the formation of curvature in the jaw-D leaf shape"—appears to conflate developmental integrity with curvature causation. The formation of stomata and petioles does not directly cause or explain curvature and this sentence should be rephrased to avoid implying causality.

We agree that this sentence is not supported by the preceding sentences, so we have removed it.

2. Lines 165-170: The authors state that cell number reaches a plateau after 6 DAS. This interpretation is not supported by Figure 1E, which shows a continued, approximately linear increase. Please adjust the text and/or the figure caption accordingly, or modify the data visualization if a true plateau exists.

Since the plateau is not clear and is not important for the conclusions of the paper, we have simplified the statement to merely say “Cell number also increased throughout the experiment (Fig 1E) [1].”

3. Lines 537-539: Please spell out "GRFs" at first mention to ensure clarity for readers unfamiliar with the abbreviation.

We have added “*GROWTH-REGULATING FSCTORs* (*GRFs*)”

4. Figure 1

- Scale bars for panels A and B are referenced only under (F); they should be specified under (A, B) already.

We moved the scale bar and sample size to A.

- The authors mention that curvature becomes pronounced after 6 DAS, but none of the Fig. 1 plots make this transition evident. Given the availability of 2.5D imaging data, could a quantitative curvature metric be derived and plotted over time to support this statement?

The curvature is quantified in Figure 2 and Supplemental Figure S8, showing this transition. Here we are referring to the appearance of curvature shown in Figure 1A, B and the associated Video S5.

5. Figures 3 and 4: Figures 3 and 4 form the core of the argument about the basipetal gradient. While the text is convincing, the quantitative aspects are difficult to appreciate from Figure 3A-D, which are mainly qualitative. Only Figures 4A-B and 4E-F clearly show gradient magnitudes. Figure 3E is particularly challenging to interpret, as no clear clustering or density trends are visible. Consider adding quantitative overlays (e.g., gradient plots or local growth vectors) or using color scales to better illustrate the data. The placement of arrows in Figure 3 should ideally be derived from quantitative values to avoid appearing arbitrary.

Figure 3A-D are heat maps which report quantitative data of measured growth rates and division rates for each cell as a color output. Since we do not have space in Figure 3 to graph these quantitative data, they are graphed in Figure 4 and Supplemental Figure S10.

We agree that Figure 3E was difficult to interpret, so we have removed it. The underlying data is still available in Supplemental Figure 9 for individual leaves, which is clearer and is sufficient to display the spatial aspect of the data. Again, this is just another way of visualizing the same data that is already shown in Figure 3, Figure 4 and Supplemental Figure S10.

6. Figure 5E-F and lines 331-339 (Growth heterogeneity): The discussion of reduced growth heterogeneity in jaw-D is currently underdeveloped. It remains unclear how decreased variability contributes to the curvature phenotype. Expanding on this connection—or clarifying if this effect is secondary—would help clarify the mechanistic link between growth coordination and mechanical stability.

We thank the reviewer for highlighting that the link between growth heterogeneity and curvature was not sufficiently explained in the original manuscript. We have revised the corresponding paragraph to clarify the mechanistic interpretation.

In the revised text, we explain that reduced growth heterogeneity in *jaw-D* should not be interpreted as directly causing curvature. Instead, the key factor is the spatial structure and orientation of growth gradients, rather than the overall magnitude of variability. The cellular Fourier analysis shows that large-scale heterogeneity is reduced in *jaw-D* primarily because the basipetal growth gradient is flattened, leading to more uniform growth along the proximal–distal axis. At the same time, our measurements reveal a relative enhancement of mediolateral growth differences, particularly between the midrib and the adjacent blade.

We now explicitly discuss that these two effects have different mechanical consequences. In wild type, the strong basipetal gradient generates heterogeneous growth that can be accommodated without mechanical conflict. In contrast, the enhanced mediolateral gradient in *jaw-D* introduces spatial incompatibilities across the width of the leaf, which our modeling later demonstrates can generate curvature. Thus, the Fourier analysis primarily serves to confirm the two major growth defects identified in the study and to highlight that curvature arises from the orientation of differential growth, rather than from increased variability per se.

Reviewer #2: This interesting paper provides novel insights into the growth dynamics that enable leaves to stay flat, and how this process is perturbed in a particular key mutant. This goes substantially beyond the current 'gene-network' understanding to put the organ-scale leaf growth into a multicellular framework. The paper presents careful image analysis, and analysis of growth kinematics to produce detailed data, and the hypotheses presented are further tested via mechanistic modelling. I believe this will be of interest to readers of PloS Biology.

Thank you for the positive comments.

I have several comments/suggestions that the authors should address:

The finding that the jaw_D phenotype partly arises from the leaf edges growing faster than the midrib is not that surprising given the observed leaf shapes. I think this might be a natural guess. However, can the authors use the modelling to gain more understanding of whether this is the only possible mechanism. Are there other possibly scenarios that could explain the ruffling? Would the ruffling occur if the only difference between the two lines was the growth differences across the leaf width, or are the longitudinal variations enabling the ruffling to be

more pronounced or somehow more robust. Presumably the ruffing does not occur if only the longitudinal growth rates are changed, but did the authors check this with their model.

Thanks for the suggestion to explore more growth patterns and the curvatures generated with the model. We have added a new results section with six simulations of additional growth patterns, Figure 9, and Videos S6-S13. First, as suggested, we removed the basipetal growth gradient from the simulation for both wild type and *jaw-D* and found that it had no effect on curvature: wild type remained flat and *jaw-D* remained equally curved (Fig 9C-D; Videos S8-S9). Second, we tested whether mediolateral growth that is uncoordinated in the proximal-distal direction could also produce curvature. We tried two growth patterns. Slow mediolateral growth at the tip of the leaf caused the simulated leaf to dome (Fig 9E and Video S10). Slow mediolateral growth in the middle of the leaf causes the simulated leaf to twist (Fig 9F and Video S11). Then we combined growth that is uncoordinated across both axes. With equal amounts of uncoordinated growth, the simulated leaf still twisted (Fig 9G and Video S12). With two times as much uncoordinated mediolateral growth as proximal distal growth, the simulated leaf formed a saddle shape (Figure 9H and Video S13).

Some of the discussions reminded me of the work of Beverley Glover and Edwige Moyroud in Cambridge who have considered the development of petal shapes with ridges that enable iridescence (in this case a rippling that is beneficial to the plant for attracting pollinators). I wondered if the authors considered this relevant to the introduction.

Good point. We have added a sentence in the introduction to highlight the connection with this work: “At a smaller scale, mechanical stress causes the cuticle at the base of the hibiscus petal to buckle, forming micro-ridges that generate iridescence (Antoniou Kourounioti et al., 2012; Airoldi et al, 2021; Moyroud et al., 2022).”

I found the modelling part (lines 264-278) very confusing. My understanding is that plant cells grow exponentially with a relative growth rate, as if not, then cells would have an abrupt change in growth rate after a division (making the tissue-scale growth unviable). Is this not the case in the leaf? The text suggests that the leaf dynamics exit an initial exponential phrase, and the figure suggests the growth rate is in 'a.u.'. Is this a cell-based model, or a continuum model? How is the growth rate included? Is the Proximal-Distal position (in Fig 4C) simply distance from the leaf base? Is this the graph of the growth rate at t=0, how does this change over time?

We apologize for the confusion and have modified both the figure and the model text to explain these concepts. Our model is cell-based, where each cell along the spatio-temporal axis has a probability to divide in a given time step (one day). We refer to this probability as the growth rate, and its spatial profile along the Proximal-Distal axis (distance from the leaf base) changes with developmental time. As correctly pointed out by the reviewer, the older figure version just showed the growth rate at a fixed time point. The updated figure now shows the spatial growth profile at different developmental time points for both genotypes. Also, the units a.u. was incorrect and has been removed.

The text is very unclear about explaining the fact that there is a spatial gradient in the growth rate that is moves with time, so that the maximum gradient moves towards the leaf base. (Or at least this is what I think it's trying to explain!) The term 'basipetal growth gradient' would naturally refer to the spatial gradient, but when first introduced (line 231) it is said this is 'progressing from the tip to the base' implying a dynamic movement with time. Around line 383 it's clearly talking about a basipetal wave, or (line 386) a growth front. But then line 417 'the flattening of the basipetal wave of maturation - presumably some dynamic change in the spatial gradient, or is this is a flattening of the way speed. Then line 487, 'slowing of the growth gradient towards the tip' does this mean reducing the spatial gradient, or slowing the movement of the growth gradient. There are other examples I could point to, but hopefully this gives the general point. Please could the authors carefully consider each reference to the spatial gradient and it's dynamic change and ensure this is as clear as possible. Maybe a schematic as an additional figure panel would help.

We agree that the spatial and temporal changes in growth during wild type development are complex and that we did not explain it clearly. We think the concept of basipetal growth gradient is so important that we have extended our description in the first paragraph of the introduction: "In many species, including *Arabidopsis thaliana*, leaf development is characterized by a dynamic transition from a rapid to slow relative growth rate known as the *basipetal growth gradient* [7,11–13]. As the leaf expands, the relative growth rate first slows at the distal tip and this zone of reduced growth progressively advances toward the proximal base. The decline in growth coincides with a basipetally moving wave of cellular differentiation and exit from the cell cycle (Donnelly et al., 1999). Over developmental time, two coordinated processes occur simultaneously: (1) the wave of growth slowing and differentiation progresses from tip to base, and (2) overall growth rates gradually decrease throughout the leaf, including within the initially fast-growing basal region (Remler, 2012). At any given time point, these dynamics produce a spatial gradient in which cells at the distal tip grow more slowly than cells nearer the base. We therefore use the term basipetal growth gradient to refer to this integrated spatiotemporal pattern of growth deceleration and differentiation during leaf development."

Throughout the rest of the paper, we have unified our language and explained clearly how the growth pattern is affected in *jaw-D*. We have included a representation of the basipetal growth gradient and how it changes in time as the leaf elongates in the new Figure 4C.

Smaller comments/typos:

Line 137, I think organ-scale measurements should be hyphenated.

Fixed.

Lines 160-174 describes cell density - is this the average cell density across all the leaf?

Yes, cell density = # cells / sum(cell areas) across all the leaf. We have clarified that in the figure legend.

Line 197, why is p given to 7 sf?

Good point. We have reduced it to 3 significant figures, matching the measurement.

Line 198, says that jaw-D are more sensitive to perturbations - this felt quite vague - what sort of perturbations are being referred to here.

We have removed this sentence.

Line 337 prevalent at 4-6 (DAS?)

Yes, thanks for catching that omission.

Line 353, 'confirming the basipetal growth gradient' Given this is an analysis of the same dataset, I think the word 'consistent with' would be a fairer interpretation.

We have changed this as suggested, thanks.
