# Supplementary material for: Growth-rate coordination across the width of a leaf preserves its flatness
Source: PLoS Biol. 2026 Sep 8;24(9):e3003993. doi: 10.1371/journal.pbio.3003993 (PMC13585336; doi:10.1371/journal.pbio.3003993)
Supplement: Supplementary file 3 [file pbio.3003993.s034.docx]

**Response to editor and reviews:**

Thanks to all for the helpful comments and suggestions. We have addressed them all as described below.

**Editor:**

a) Please make the data deposited at OSF and at GitHub publicly available at this stage. For example, we need data on Fig. 1C-F, H-K; Fig. 2A-G; Fig. 3C-E; Fig. 6B, D; Fig. S7A-G; Fig. S8A, B; Fig. S9; Fig. S10A-D; Fig. S11A, B; Fig. S12B, D, E-H; Fig. S13A-D and Fig. S14A-H

We have made all of the data and code publicly available on osf.io in two data repositories with DOIs: <https://doi.org/10.17605/OSF.IO/D7X3Y> and <https://doi.org/10.17605/OSF.IO/YTVW9>. In addition to the meshes, we have provided the numerical data. Much of this numerical data is in large data files that provide all of the different characteristics of each cell in a specific sample and time point. The graphs in the figures were made directly from these large data files using code in R. We have provided both the underlying data files and the scripts to assemble the graphs from these large data files. The R code files are labeled with figure and supplementary figure number.

Note that some of the exact figure panels cited above do not exist, such as Figure 2 C-G (Figure 2 only has A-B). Therefore, we have gone through all figures and addressed this point, not just the specific figure panels listed here.

b) Many thanks for providing the underlying data and code in Github. However, because Github depositions can be readily changed or deleted, please make a permanent DOI’d copy (e.g. in Zenodo - see instructions below my signature).

We have moved the code to our osf.io repository where it has a doi together with the data: <https://doi.org/10.17605/OSF.IO/YTVW9>. Each part is labeled by figure number and panel.

c) Please clearly cite the location of the data in all relevant main and supplementary figure legends. For example, you can add: “The data underlying this figure can be found at [doi.org/10.xxx/zenodo.xxxxx](http://doi.org/10.xxx/zenodo.xxxxx).” We understand that this may appear repetitive, but it helps make the figures and their legends more self-contained.Please also make sure to address the following data and other policy-related requests.

We have added a statement at the end of each figure, supplementary figure, and video legend stating where the data and code can be found.

d) In order to make the Title more specific and appealing, please change it to "Growth-rate coordination across the width of a leaf preserves its flatness"

We like the new title and have change it as suggested. We have also changed the running title to the same thing.

**Technical editors:**

The technical checks asked us to remove the screenshots from videos S1 and S2 due to copyright reasons.

We have cropped these videos to remove the parts of the screen capture and only leave the scientific images.

**Reviewer comments:**

Reviewer #1: Thank you to the authors for addressing my comments and clarifying the points I raised. I appreciate the effort that has gone into improving the manuscript, and I believe the revisions have strengthened it considerably. I have no further requests or concerns.

Thank you.

Reviewer #2: The authors have addressed my suggestions very thoroughly, especially in adding the extra simulations showing the predictions with alternative growth patterns. This is an excellent paper, and I think it will be well received by the field.

Thank you.
