# Supplementary material for: Growth-rate coordination across the width of a leaf preserves its flatness
Source: PLoS Biol. 2026 Sep 8;24(9):e3003993. doi: 10.1371/journal.pbio.3003993 (PMC13585336; doi:10.1371/journal.pbio.3003993)
Supplement: Peer review history

# Editor report: Decision Letter 0

**DOI:** 10.1371/journal.pbio.3003993.r001

**Authors:** Ankiit Ahluwalia

14 Oct 2025

Dear Dr Roeder,

Thank you for submitting your manuscript entitled "Cell growth rates coordinate across the width of the leaf to remain flat" for consideration as a Research Article by PLOS Biology.

Your manuscript has now been evaluated by the PLOS Biology editorial staff [as well as by an academic editor with relevant expertise] and I am writing to let you know that we would like to send your submission out for external peer review.

However, before we can send your manuscript to reviewers, we need you to complete your submission by providing the metadata that is required for full assessment. To this end, please login to Editorial Manager where you will find the paper in the 'Submissions Needing Revisions' folder on your homepage. Please click 'Revise Submission' from the Action Links and complete all additional questions in the submission questionnaire.

Once your full submission is complete, your paper will undergo a series of checks in preparation for peer review. After your manuscript has passed the checks it will be sent out for review. To provide the metadata for your submission, please Login to Editorial Manager (https://www.editorialmanager.com/pbiology) within two working days, i.e. by Oct 16 2025 11:59PM.

If your manuscript has been previously peer-reviewed at another journal, PLOS Biology is willing to work with those reviews in order to avoid re-starting the process. Submission of the previous reviews is entirely optional and our ability to use them effectively will depend on the willingness of the previous journal to confirm the content of the reports and share the reviewer identities. Please note that we reserve the right to invite additional reviewers if we consider that additional/independent reviewers are needed, although we aim to avoid this as far as possible. In our experience, working with previous reviews does save time.

If you would like us to consider previous reviewer reports, please edit your cover letter to let us know and include the name of the journal where the work was previously considered and the manuscript ID it was given. In addition, please upload a response to the reviews as a 'Prior Peer Review' file type, which should include the reports in full and a point-by-point reply detailing how you have or plan to address the reviewers' concerns.

During the process of completing your manuscript submission, you will be invited to opt-in to posting your pre-review manuscript as a bioRxiv preprint. Visit http://journals.plos.org/plosbiology/s/preprints for full details. If you consent to posting your current manuscript as a preprint, please upload a single Preprint PDF.

Feel free to email us at plosbiology@plos.org if you have any queries relating to your submission.

Kind regards,

Ankiit

Ankiit Ahluwalia,

PLOS Biology

aahluwalia@plos.org

# Peer review: Decision Letter 1

**DOI:** 10.1371/journal.pbio.3003993.r002

**Authors:** Ankiit Ahluwalia

18 Dec 2025

Dear Dr Roeder,

Thank you for your patience while your manuscript "Cell growth rates coordinate across the width of the leaf to remain flat" was peer-reviewed at PLOS Biology. It has now been evaluated by the PLOS Biology editors, an Academic Editor with relevant expertise, and by several independent reviewers.

In light of the reviews, which you will find at the end of this email, we would like to invite you to revise the work to thoroughly address the reviewers' reports.

You'll see that both reviewers are positive and find the study interesting and worth pursuing, but they also raise several points that would need to be addressed before we can consider the manuscript for publication. Reviewer1 has two concerns regarding growth at the abaxial surface, which should be considered, and that experimentally determined jaw-D growth data should be added rather than a simplified constant function in the 1D model. Reviewer2 asks for several clarifications on the modelling for improvement and also on the definition of growth gradient moving from tip to base and time involvement.

After discussing these comments with the Academic Editor and the rest of the team, we have decided to invite a revision that should address all the points raised by the reviewers.

Given the extent of revision needed, we cannot make a decision about publication until we have seen the revised manuscript and your response to the reviewers' comments. Your revised manuscript is likely to be sent for further evaluation by all or a subset of the reviewers.

In addition to these revisions, you will need to complete some formatting changes, which you will receive in a follow up email. A member of our team will be in touch with a set of requests shortly.

We expect to receive your revised manuscript within 3 months. Please email us (plosbiology@plos.org) if you have any questions or concerns, or would like to request an extension.

At this stage, your manuscript remains formally under active consideration at our journal; please notify us by email if you do not intend to submit a revision so that we may withdraw it.

**IMPORTANT - SUBMITTING YOUR REVISION**

Your revisions should address the specific points made by each reviewer. Please submit the following files along with your revised manuscript:

1. A 'Response to Reviewers' file - this should detail your responses to the editorial requests, present a point-by-point response to all of the reviewers' comments, and indicate the changes made to the manuscript.

*NOTE: In your point-by-point response to the reviewers, please provide the full context of each review. Do not selectively quote paragraphs or sentences to reply to. The entire set of reviewer comments should be present in full and each specific point should be responded to individually, point by point.

You should also cite any additional relevant literature that has been published since the original submission and mention any additional citations in your response.

2. In addition to a clean copy of the manuscript, please also upload a 'track-changes' version of your manuscript that specifies the edits made. This should be uploaded as a "Revised Article with Changes Highlighted" file type.

*Re-submission Checklist*

When you are ready to resubmit your revised manuscript, please refer to this re-submission checklist: https://plos.io/Biology_Checklist

To submit a revised version of your manuscript, please go to https://www.editorialmanager.com/pbiology/ and log in as an Author. Click the link labelled 'Submissions Needing Revision' where you will find your submission record.

Please make sure to read the following important policies and guidelines while preparing your revision:

*Published Peer Review*

Please note while forming your response, if your article is accepted, you may have the opportunity to make the peer review history publicly available. The record will include editor decision letters (with reviews) and your responses to reviewer comments. If eligible, we will contact you to opt in or out. Please see here for more details:

https://blogs.plos.org/plos/2019/05/plos-journals-now-open-for-published-peer-review/

*PLOS Data Policy*

Please note that as a condition of publication PLOS' data policy (http://journals.plos.org/plosbiology/s/data-availability) requires that you make available all data used to draw the conclusions arrived at in your manuscript. If you have not already done so, you must include any data used in your manuscript either in appropriate repositories, within the body of the manuscript, or as supporting information (N.B. this includes any numerical values that were used to generate graphs, histograms etc.). For an example see here: http://www.plosbiology.org/article/info%3Adoi%2F10.1371%2Fjournal.pbio.1001908#s5

*Blot and Gel Data Policy*

We require the original, uncropped and minimally adjusted images supporting all blot and gel results reported in an article's figures or Supporting Information files. We will require these files before a manuscript can be accepted so please prepare them now, if you have not already uploaded them. Please carefully read our guidelines for how to prepare and upload this data: https://journals.plos.org/plosbiology/s/figures#loc-blot-and-gel-reporting-requirements

*Protocols deposition*

To enhance the reproducibility of your results, we recommend that if applicable you deposit your laboratory protocols in protocols.io, where a protocol can be assigned its own identifier (DOI) such that it can be cited independently in the future. Additionally, PLOS ONE offers an option for publishing peer-reviewed Lab Protocol articles, which describe protocols hosted on protocols.io. Read more information on sharing protocols at https://plos.org/protocols?utm_medium=editorial-email&utm_source=authorletters&utm_campaign=protocols

Thank you again for your submission to our journal. We hope that our editorial process has been constructive thus far, and we welcome your feedback at any time. Please don't hesitate to contact us if you have any questions or comments.

Sincerely,

Ankiit

Ankiit Ahluwalia,

PLOS Biology

aahluwalia@plos.org

------------------------------------

REVIEWS:

Reviewer #1: This manuscript investigates the cellular basis of leaf flatness by comparing developing wild-type (Arabidopsis thaliana) leaves with the curved and ruffled leaves of the jaw-D mutant. Through time-lapse live imaging (3-8 days after sowing) combined with finite element modeling, the authors track cellular growth, division, and maturation dynamics. The jaw-D mutant overexpresses miR319, which represses TCP transcription factors and delays leaf maturation.

The study identifies two principal disruptions in jaw-D growth patterns:

(i) the canonical basipetal growth-rate gradient—typically characterized by slow growth at the tip and progressively faster growth toward the base—is flattened, resulting in a more uniform, moderate growth along the entire proximal-distal axis; and

(ii) growth across the width of the leaf (medial-lateral axis) becomes non-uniform, with slower expansion in the midrib region relative to the adjacent blade.

The second point, the failure of mediolateral growth coordination, represents the most critical and unexpected discovery of the study and provides a compelling explanation for the curvature phenotypes observed in jaw-D. The modeling results elegantly demonstrate that proximal-distal growth differences alone cannot account for the curvature, thus revealing that leaf flatness requires coordinated growth across the entire width of the tissue to prevent internal mechanical conflicts.

Furthermore, the authors connect this loss of mediolateral coordination to delayed pavement cell maturation, which compromises the mechanical stability of the tissue. The normal development of stomata serves as an effective internal control, underscoring that the flatness instability arises specifically from the pavement cell domain (and thus mechanics!) rather than from general developmental defects.

Taken together, the integration of live imaging and modeling in this study is truly exceptional—offering a mechanistic depth that is rarely achieved in plant morphogenesis research.

General assessment

This work advances our understanding of how organ flatness is maintained. Whereas previous models of leaf curvature focused primarily on growth differences between the adaxial and abaxial surfaces, this study reveals that coordination of cell expansion across the width of the leaf is equally critical. The authors' demonstration that differential mediolateral growth alone is sufficient to reproduce curvature represents a conceptual shift in how we think about planar organ development.

The study also establishes clear parallels with biomechanical systems in other organisms (e.g., Drosophila wing discs, kelp fronds, animal epithelia) where local growth mismatches generate shape complexity. Overall, the manuscript is well written, methodologically rigorous, and conceptually important. I strongly support publication after revision, provided that the points below are addressed.

Major comments

1. Adaxial-abaxial comparison: In the Introduction, the authors correctly note that leaf curvature is typically attributed to differential growth between adaxial and abaxial tissues. However, the study quantifies growth exclusively on the abaxial side. Have the authors performed, or at least considered, comparable long-term imaging of the adaxial surface? Such data would be essential to determine whether the same growth irregularities (flattened basipetal gradient and non-uniform medial-lateral growth) also occur adaxially. Without this information, the mechanistic link between side-specific growth and curvature remains incomplete. While this limitation does not undermine the main aim of the manuscript, it should be discussed more openly.

2. Modeling of leaf length (proximal-distal axis): The 1D model used to simulate leaf length differences between WT and jaw-D approximates the jaw-D growth profile as a constant moderate growth rate along the proximal-distal axis, despite the availability of more detailed measurements. Since the model concludes that "different proximal-distal growth patterns can yield similar leaf lengths," it would be more convincing to incorporate the experimentally determined jaw-D growth data rather than a simplified constant function. Otherwise, the conclusion risks appearing speculative or selectively supported.

Importantly, the authors should highlight that this and other modeling exercises demonstrate that proximal-distal growth defects alone cannot explain the curvature phenotype—thereby motivating their subsequent focus on mediolateral coordination failure as the physical driver of curvature. Making this logical connection more explicit would greatly strengthen the narrative coherence of the manuscript.

Minor comments

1. Lines 145-157: The authors show that stomatal initiation and petiole formation occur normally in jaw-D, indicating that major developmental programs remain intact. However, the following statement—"Thus, in the first 8 days of leaf development, we have captured key changes in cellular growth that cause the formation of curvature in the jaw-D leaf shape"—appears to conflate developmental integrity with curvature causation. The formation of stomata and petioles does not directly cause or explain curvature and this sentence should be rephrased to avoid implying causality.

2. Lines 165-170: The authors state that cell number reaches a plateau after 6 DAS. This interpretation is not supported by Figure 1E, which shows a continued, approximately linear increase. Please adjust the text and/or the figure caption accordingly, or modify the data visualization if a true plateau exists.

3. Lines 537-539: Please spell out "GRFs" at first mention to ensure clarity for readers unfamiliar with the abbreviation.

4. Figure 1

- Scale bars for panels A and B are referenced only under (F); they should be specified under (A, B) already.

- The authors mention that curvature becomes pronounced after 6 DAS, but none of the Fig. 1 plots make this transition evident. Given the availability of 2.5D imaging data, could a quantitative curvature metric be derived and plotted over time to support this statement?

5. Figures 3 and 4: Figures 3 and 4 form the core of the argument about the basipetal gradient. While the text is convincing, the quantitative aspects are difficult to appreciate from Figure 3A-D, which are mainly qualitative. Only Figures 4A-B and 4E-F clearly show gradient magnitudes. Figure 3E is particularly challenging to interpret, as no clear clustering or density trends are visible. Consider adding quantitative overlays (e.g., gradient plots or local growth vectors) or using color scales to better illustrate the data. The placement of arrows in Figure 3 should ideally be derived from quantitative values to avoid appearing arbitrary.

6. Figure 5E-F and lines 331-339 (Growth heterogeneity): The discussion of reduced growth heterogeneity in jaw-D is currently underdeveloped. It remains unclear how decreased variability contributes to the curvature phenotype. Expanding on this connection—or clarifying if this effect is secondary—would help clarify the mechanistic link between growth coordination and mechanical stability.

Reviewer #2: This interesting paper provides novel insights into the growth dynamics that enable leaves to stay flat, and how this process is perturbed in a particular key mutant. This goes substantially beyond the current 'gene-network' understanding to put the organ-scale leaf growth into a multicellular framework. The paper presents careful image analysis, and analysis of growth kinematics to produce detailed data, and the hypotheses presented are further tested via mechanistic modelling. I believe this will be of interest to readers of PloS Biology.

I have several comments/suggestions that the authors should address:

The finding that the jaw_D phenotype partly arises from the leaf edges growing faster than the midrib is not that surprising given the observed leaf shapes. I think this might be a natural guess. However, can the authors use the modelling to gain more understanding of whether this is the only possible mechanism. Are there other possibly scenarios that could explain the ruffling? Would the ruffling occur if the only difference between the two lines was the growth differences across the leaf width, or are the longitudinal variations enabling the ruffling to be more pronounced or somehow more robust. Presumably the ruffing does not occur if only the longitudinal growth rates are changed, but did the authors check this with their model.

Some of the discussions reminded me of the work of Beverley Glover and Edwige Moyroud in Cambridge who have considered the development of petal shapes with ridges that enable iridescence (in this case a rippling that is beneficial to the plant for attracting pollinators). I wondered if the authors considered this relevant to the introduction.

I found the modelling part (lines 264-278) very confusing. My understanding is that plant cells grow exponentially with a relative growth rate, as if not, then cells would have an abrupt change in growth rate after a division (making the tissue-scale growth unviable). Is this not the case in the leaf? The text suggests that the leaf dynamics exit an initial exponential phrase, and the figure suggests the growth rate is in 'a.u.'. Is this a cell-based model, or a continuum model? How is the growth rate included? Is the Proximal-Distal position (in Fig 4C) simply distance from the leaf base? Is this the graph of the growth rate at t=0, how does this change over time?

The text is very unclear about explaining the fact that there is a spatial gradient in the growth rate that is moves with time, so that the maximum gradient moves towards the leaf base. (Or at least this is what I think it's trying to explain!) The term 'basipetal growth gradient' would naturally refer to the spatial gradient, but when first introduced (line 231) it is said this is 'progressing from the tip to the base' implying a dynamic movement with time. Around line 383 it's clearly talking about a basipetal wave, or (line 386) a growth front. But then line 417 'the flattening of the basipetal wave of maturation - presumably some dynamic change in the spatial gradient, or is this is a flattening of the way speed. Then line 487, 'slowing of the growth gradient towards the tip' does this mean reducing the spatial gradient, or slowing the movement of the growth gradient. There are other examples I could point to, but hopefully this gives the general point. Please could the authors carefully consider each reference to the spatial gradient and it's dynamic change and ensure this is as clear as possible. Maybe a schematic as an additional figure panel would help.

Smaller comments/typos:

Line 137, I think organ-scale measurements should be hyphenated.

Lines 160-174 describes cell density - is this the average cell density across all the leaf?

Line 197, why is p given to 7 sf?

Line 198, says that jaw-D are more sensitive to perturbations - this felt quite vague - what sort of perturbations are being referred to here.

Line 337 prevalent at 4-6 (DAS?)

Line 353, 'confirming the basipetal growth gradient' Given this is an analysis of the same dataset, I think the word 'consistent with' would be a fairer interpretation.

# Author comment: Author response to Decision Letter 2

**DOI:** 10.1371/journal.pbio.3003993.r003

1 Jun 2026

Attachment Submitted filename: jaw-D PLOS Bio response to reviewers.pdf

# Peer review: Decision Letter 2

**DOI:** 10.1371/journal.pbio.3003993.r004

**Authors:** Ankiit Ahluwalia

16 Jul 2026

Dear Dr Roeder,

Thank you for your patience while we considered your revised manuscript "Cell growth rates coordinate across the width of the leaf to remain flat" for publication as a Research Article at PLOS Biology. This revised version of your manuscript has been evaluated by the PLOS Biology editors, the Academic Editor, and the original reviewers.

Based on the reviews, we are likely to accept this manuscript for publication, provided you satisfactorily address the remaining points (below):

IMPORTANT - please attend to the following:

a) Please make the data deposited at OSF and at GitHub publicly available at this stage. For example, we need data on Fig. 1C-F, H-K; Fig. 2A-G; Fig. 3C-E; Fig. 6B, D; Fig. S7A-G; Fig. S8A, B; Fig. S9; Fig. S10A-D; Fig. S11A, B; Fig. S12B, D, E-H; Fig. S13A-D and Fig. S14A-H

b) Many thanks for providing the underlying data and code in Github. However, because Github depositions can be readily changed or deleted, please make a permanent DOI’d copy (e.g. in Zenodo - see instructions below my signature).

c) Please clearly cite the location of the data in all relevant main and supplementary figure legends. For example, you can add: “The data underlying this figure can be found at doi.org/10.xxx/zenodo.xxxxx.” We understand that this may appear repetitive, but it helps make the figures and their legends more self-contained.Please also make sure to address the following data and other policy-related requests.

d) In order to make the Title more specific and appealing, please change it to "Growth-rate coordination across the width of a leaf preserves its flatness"

As you address these items, please take this last chance to review your reference list to ensure that it is complete and correct. If you have cited papers that have been retracted, please include the rationale for doing so in the manuscript text, or remove these references and replace them with relevant current references. Any changes to the reference list should be mentioned in the cover letter that accompanies your revised manuscript.

In addition to these revisions, you may need to complete some formatting changes, which you will receive in a follow up email. A member of our team will be in touch with a set of requests shortly. If you do not receive a separate email within a few days, please assume that checks have been completed, and no additional changes are required.

We expect to receive your revised manuscript within two weeks.

To submit your revision, please go to https://www.editorialmanager.com/pbiology/ and log in as an Author. Click the link labelled 'Submissions Needing Revision' to find your submission record. Your revised submission must include the following:

- a cover letter that should detail your responses to any editorial requests, if applicable, and whether changes have been made to the reference list

- a Response to Reviewers file that provides a detailed response to the reviewers' comments (if applicable, if not applicable please do not delete your existing 'Response to Reviewers' file.)

- a track-changes file indicating any changes that you have made to the manuscript.

NOTE: If Supporting Information files are included with your article, note that these are not copyedited and will be published as they are submitted. Please ensure that these files are legible and of high quality (at least 300 dpi) in an easily accessible file format. For this reason, please be aware that any references listed in an SI file will not be indexed. For more information, see our Supporting Information guidelines:

https://journals.plos.org/plosbiology/s/supporting-information

*Published Peer Review History*

Please note that you may have the opportunity to make the peer review history publicly available. The record will include editor decision letters (with reviews) and your responses to reviewer comments. If eligible, we will contact you to opt in or out. Please see here for more details:

https://plos.org/published-peer-review-history/

*Press*

Should you, your institution's press office or the journal office choose to press release your paper, please ensure you have opted out of Early Article Posting on the submission form. We ask that you notify us as soon as possible if you or your institution is planning to press release the article.

*Protocols deposition*

To enhance the reproducibility of your results, we recommend that if applicable you deposit your laboratory protocols in protocols.io, where a protocol can be assigned its own identifier (DOI) such that it can be cited independently in the future. Additionally, PLOS ONE offers an option for publishing peer-reviewed Lab Protocol articles, which describe protocols hosted on protocols.io. Read more information on sharing protocols at https://plos.org/protocols?utm_medium=editorial-email&utm_source=authorletters&utm_campaign=protocols

Please do not hesitate to contact me should you have any questions.

Sincerely,

Ankiit

Ankiit Ahluwalia,

aahluwalia@plos.org

PLOS Biology

------------------------------------------------------------------------

****[DELETE AS APPROPRIATE]****

ETHICS STATEMENT:

-- Please include the full name of the IACUC/ethics committee that reviewed and approved the animal care and use protocol/permit/project license. Please also include an approval number.

-- Please include the specific national or international regulations/guidelines to which your animal care and use protocol adhered. Please note that institutional or accreditation organization guidelines (such as AAALAC) do not meet this requirement.

-- Please include information about the form of consent (written/oral) given for research involving human participants. All research involving human participants must have been approved by the authors' Institutional Review Board (IRB) or an equivalent committee, and must have been conducted according to the principles expressed in the Declaration of Helsinki.

------------------------------------------------------------------------

DATA POLICY:

You may be aware of the PLOS Data Policy, which requires that all data be made available without restriction: http://journals.plos.org/plosbiology/s/data-availability. For more information, please also see this editorial: http://dx.doi.org/10.1371/journal.pbio.1001797

Note that we do not require all raw data. Rather, we ask that all individual quantitative observations that underlie the data summarized in the figures and results of your paper be made available in one of the following forms:

1) Supplementary files (e.g., excel). Please ensure that all data files are uploaded as 'Supporting Information' and are invariably referred to (in the manuscript, figure legends, and the Description field when uploading your files) using the following format verbatim: S1 Data, S2 Data, etc. Multiple panels of a single or even several figures can be included as multiple sheets in one excel file that is saved using exactly the following convention: S1_Data.xlsx (using an underscore).

2) Deposition in a publicly available repository. Please also provide the accession code or a reviewer link so that we may view your data before publication.

Regardless of the method selected, please ensure that you provide the individual numerical values that underlie the summary data displayed in the following figure panels as they are essential for readers to assess your analysis and to reproduce it:

[Figs….]

NOTE: the numerical data provided should include all replicates AND the way in which the plotted mean and errors were derived (it should not present only the mean/average values).

Please also ensure that figure legends in your manuscript include information on where the underlying data can be found, and ensure your supplemental data file/s has a legend.

Please ensure that your Data Statement in the submission system accurately describes where your data can be found.

------------------------------------------------------------------------

APPROPRIATE DATA PRESENTATION?

(If you think the paper is not using best practice, add the following)

- Please ensure that you are using best practice for statistical reporting and data presentation. These are our guidelines https://journals.plos.org/plosbiology/s/best-practices-in-research-reporting#loc-statistical-reporting and a useful resource on data presentation https://journals.plos.org/plosbiology/article?id=10.1371/journal.pbio.1002128

- If you are reporting experiments where n ≤ 5, please plot each individual data point.

------------------------------------------------------------------------

CODE POLICY

Per journal policy, if you have generated any custom code during the course of this investigation, please make it available without restrictions. Please ensure that the code is sufficiently well documented and reusable, and that your Data Statement in the Editorial Manager submission system accurately describes where your code can be found. More information on our Code Policy, what and how to share can be found here: https://journals.plos.org/plosbiology/s/code-availability

Please note that we cannot accept sole deposition of code in GitHub, as this could be changed after publication. However, you can archive this version of your publicly available GitHub code to Zenodo. Once you do this, it will generate a DOI number, which you will need to provide in the Data Accessibility Statement (you are welcome to also provide the GitHub access information). See the process for doing this here: https://docs.github.com/en/repositories/archiving-a-github-repository/referencing-and-citing-content

------------------------------------------------------------------------

SPECIES INDICATED IN THE ABSTRACT?

- Please note that per journal policy, the model system/species studied should be clearly stated in the abstract of your manuscript.

**EDITOR, CONSIDER ALSO INCLUDING ORGANISM IN THE TITLE, IF SENSICAL/RELEVANT AND/OR TO AVOID BEING MISLEADING/OVERREACHING (not always preferred)**

------------------------------------------------------------------------

BLOT AND GEL REPORTING REQUIREMENTS:

We require the original, uncropped and minimally adjusted images supporting all blot and gel results reported in an article's figures or Supporting Information files. We will require these files before a manuscript can be accepted so please prepare and upload them now. Please carefully read our guidelines for how to prepare and upload this data: https://journals.plos.org/plosbiology/s/figures#loc-blot-and-gel-reporting-requirements

------------------------------------------------------------------------

DATA NOT SHOWN?

- Please note that per journal policy, we do not allow the mention of "data not shown", "personal communication", "manuscript in preparation" or other references to data that is not publicly available or contained within this manuscript. Please either remove mention of these data or provide figures presenting the results and the data underlying the figure(s).

------------------------------------------------------------------------

Reviewer remarks:

Reviewer #1: Thank you to the authors for addressing my comments and clarifying the points I raised. I appreciate the effort that has gone into improving the manuscript, and I believe the revisions have strengthened it considerably. I have no further requests or concerns.

Reviewer #2: The authors have addressed my suggestions very thoroughly, especially in adding the extra simulations showing the predictions with alternative growth patterns. This is an excellent paper, and I think it will be well received by the field.

# Author comment: Author response to Decision Letter 3

**DOI:** 10.1371/journal.pbio.3003993.r005

14 Aug 2026

Attachment Submitted filename: Response to editor and reviewers.docx

# Editor report: Decision Letter 3

**DOI:** 10.1371/journal.pbio.3003993.r006

**Authors:** Ankiit Ahluwalia

24 Aug 2026

Dear Dr Roeder,

Thank you for the submission of your revised Research Article "Growth-rate coordination across the width of a leaf preserves its flatness" for publication in PLOS Biology. On behalf of my colleagues and the Academic Editor, Alexander Jones, I am pleased to say that we can in principle accept your manuscript for publication, provided you address any remaining formatting and reporting issues. These will be detailed in an email you should receive within 2-3 business days from our colleagues in the journal operations team; no action is required from you until then. Please note that we will not be able to formally accept your manuscript and schedule it for publication until you have completed any requested changes.

Please take a minute to log into Editorial Manager at http://www.editorialmanager.com/pbiology/, click the "Update My Information" link at the top of the page, and update your user information to ensure an efficient production process.

PRESS

We frequently collaborate with press offices. If your institution or institutions have a press office, please notify them about your upcoming paper at this point, to enable them to help maximise its impact. If the press office is planning to promote your findings, we would be grateful if they could coordinate with biologypress@plos.org. If you have previously opted in to the early version process, we ask that you notify us immediately of any press plans so that we may opt out on your behalf.

We also ask that you take this opportunity to read our Embargo Policy regarding the discussion, promotion and media coverage of work that is yet to be published by PLOS. As your manuscript is not yet published, it is bound by the conditions of our Embargo Policy. Please be aware that this policy is in place both to ensure that any press coverage of your article is fully substantiated and to provide a direct link between such coverage and the published work. For full details of our Embargo Policy, please visit http://www.plos.org/about/media-inquiries/embargo-policy/.

Thank you again for choosing PLOS Biology for publication and supporting Open Access publishing. We look forward to publishing your study.

Sincerely,

Ankiit Ahluwalia,

PLOS Biology

aahluwalia@plos.org
